# PLK1-mediated phosphorylation of β-catenin enhances its stability and transcriptional activity for extracellular matrix remodeling in metastatic NSCLC

**DOI:** 10.7150/thno.79318

**Published:** 2023-02-05

**Authors:** Da-Eun Kim, Sol-Bi Shin, Chang-Hyeon Kim, Yeo-Bin Kim, Hyun-Ji Oh, Hyungshin Yim

**Affiliations:** 1 Department of Pharmacy, College of Pharmacy, Hanyang University, Ansan, Gyeonggi-do 15588, Korea; 2 Institute of Pharmaceutical Science and Technology, Hanyang University, Ansan, Gyeonggi-do 15588, Korea

**Keywords:** PLK1, β-catenin, AP-1, extracellular matrix, lung cancer

## Abstract

**Rationale:** β-catenin is a component for cell adhesion and a transcriptional coactivator in epithelial-mesenchymal transition (EMT). Previously we found that catalytically active PLK1 drives EMT in non-small cell lung cancer (NSCLC), upregulating extracellular matrix factors including TSG6, laminin γ2, and CD44. To understand the underlying mechanism and clinical significance of PLK1 and β-catenin in NSCLC, their relationship and function in metastatic regulation were investigated.

**Methods:** The clinical relevance between the survival rate of NSCLC patients and the expression of PLK1 and β-catenin was analyzed by a KM plot. Immunoprecipitation, kinase assay, LC-MS/MS spectrometry, and site-directed mutagenesis were performed to reveal their interaction and phosphorylation. A lentiviral doxycycline-inducible system, Transwell-based 3D culture, tail-vein injection model, confocal microscopy, and chromatin immunoprecipitation assays were used to elucidate the function of phosphorylated β-catenin in the EMT of NSCLC.

**Results:** Clinical analysis revealed that the high expression of *CTNNB1/PLK1* was inversely correlated with the survival rates of 1,292 NSCLC patients, especially in metastatic NSCLC. In TGF-β-induced or active PLK1-driven EMT, β-catenin, PLK1, TSG6, laminin γ2, and CD44 were concurrently upregulated. β-catenin is a binding partner of PLK1 in TGF-β-induced EMT and is phosphorylated at S311. Phosphomimetic β-catenin promotes cell motility, invasiveness of NSCLC cells, and metastasis in a tail-vein injection mouse model. Its upregulated stability by phosphorylation enhances transcriptional activity through nuclear translocation for the expression of laminin γ2, CD44, and c-Jun, therefore enhancing PLK1 expression by AP-1.

**Conclusions:** Our findings provide evidence for the critical role of the PLK1/β-catenin/AP-1 axis in metastatic NSCLC, implying that β-catenin and PLK1 may serve as a molecular target and prognostic indicator of the therapeutic response in metastatic NSCLC patients.

## Introduction

Accumulating evidence indicates that the epithelial-mesenchymal transition (EMT), an initial event in metastasis for acquiring invasiveness, is driven by a variety of growth factors such as transforming growth factor (TGF)-β 1, Wnt 2, and EGF 3, 4. As a central component of Wnt signals, β-catenin functions as a component of the cadherin/catenin complex for cell adhesion 2 and a transcriptional coactivator in TGF-β-induced EMT and metastasis 5. During EMT, a tyrosine kinase c-Src phosphorylates β-catenin, causing it to lose its universal binding to E-cadherin 2, 6, 7. Cytosolic β-catenin separated from E-cadherin translocates into the nucleus and promotes gene expression for EMT by binding with T-cell factor (TCF)/lymphocyte enhancer factor (LEF) 5, 6. The stability of β-catenin is regulated by phosphorylation and degradation depending on Wnt signaling 8, 9. The presence of Wnt prevents the degradation of β-catenin. The accumulation of β-catenin results in its entry into the nucleus and binding with the transcription factor TCF/LEF 2, 10. However, in the absence of Wnt, the N-terminus of β-catenin is phosphorylated by GSK3β and CK1α, which promotes its degradation mediated by β-TrCP 8, 9. In contrast to the degradation of β-catenin through N-terminal phosphorylation by GSK3β and CK1α, the C-terminal phosphorylation of β-catenin by protein kinase A at Ser675 11, AKT at Ser552 12, and JNK2 at Ser191 and Ser605 13 are related with its stabilization and nuclear accumulation, which results in β-catenin-mediated transcriptional activation. The phosphorylation at Ser60 by PLK1 functions in mitosis for the completion of cytokinesis 14. Additional phosphorylation by PLK1 at Ser718 during mitosis 15 was also reported, although their function in phosphorylation is not clearly understood.

PLK1 functions as a mitotic protein kinase and is a proliferation factor that is a favorable therapeutic target for cancer treatment 16, 17. PLK1 is proposed as an important driver in EMT and metastasis 18 in gastric cancer 19, lung cancer 20, pancreatic cancer 21, and kidney cancer 22. Catalytically active PLK1 promotes metastasis in non-small cell lung cancer (NSCLC) through the amplification of TGF-β signaling 20 and shows a high frequency of extracellular matrix (ECM)-adhesion, immune system, and TGF-β signaling in the KEGG pathway analysis of transcriptome profiles and highly expressed ECM adhesion-related genes, including *TNFAIP6*, *LAMC2*, and *LCE3D*, which are the top three genes in microarray analysis 19. *LAMC2*, a direct transcriptional target of β-catenin, is an important metastatic indicator of lung adenocarcinoma 23, 24. TSG6 encoded by *TNFAIP6* is a hyaluronan (HA) binding protein that is involved in ECM stability during EMT and cell migration. HA is a central factor in the EMT of renal tubular epithelial cells through a CD44-dependent manner 25, 26. CD44, another important transcriptional target of β-catenin, is reported as a cancer stem cell marker and a mediator of TGF-β-induced metastasis 27, 28. Although *TNFAIP6*, *LAMC2*, and *CD44* are highly upregulated in active PLK1-driven or TGF-β-induced metastasis 20, the precise mechanism and correlation between these factors in metastasis still have to be investigated. Since it is known that *LAMC2* and *CD44* are the transcriptional target genes of β-catenin 24, 28, we hypothesized that β-catenin can regulate this event as a transcription factor for expressing *LAMC2* and *CD44* in PLK1-driven EMT*.* Upregulation of CD44 by β-catenin would function through interacting with hyaluronate and TSG6 25, 29. Additionally, c-Jun, a component of AP-1 30, is known as a transcriptional target and binding partner of β-catenin in colorectal cancer 31 and is a possible transcriptional factor for PLK1 expression 32. For these reasons, we investigated the relationship between PLK1 and β-catenin for the expression of ECM adhesion-related factors including *TNFAIP6*, *LAMC2*, and *CD44* during the EMT of NSCLC. Here, we demonstrate that PLK1 regulates the stability of β-catenin through phosphorylation during EMT in NSCLC and facilitates its translocation in the nucleus where it regulates the expression of *CD44*, *LAMC2*, and *JUN* to accelerate malignancy, including metastasis. Consequently, the formation of the AP-1 complex by the expression of* JUN* triggered by stabilized β-catenin facilitates PLK1-mediated EMT in metastatic NSCLC.

## Materials and Methods

### Bioinformatics analysis

NSCLC patient data were obtained from an online database (www.kmplot.com) following a previous report 33. All cancer patients in the database were identified from the Gene Expression Omnibus (http://www.ncbi.nlm.nih.gov/geo/) or the Cancer Genome Atlas (http://cancergenome.nih.gov). The database, which was established using gene expression data and survival information from 1,292 patients, was used to establish the clinical relevance of β-catenin and PLK1 expression to the survival of patients, after excluding biased arrays. The samples were split into high and low groups using the median expression of each factor. Hazard ratios (HRs) with 95% confidence intervals and log-rank *P* were calculated according to the online formulas provided by each database. A log-rank *P* value of <0.05 was considered statistically significant. An HR is the ratio of the hazard rates that correspond to the conditions described by two levels of an explanatory variable in a survival analysis as shown in Table S1. Prediction of transcriptional factors in the ChIP-Seq database was performed using Appyter (https://appyters.maayanlab.cloud/#/ ChEA3_Appyter).

### Cell culture and treatment

NSCLC A549 and NCI-H460 cells were purchased from the KCLB (KCLB; Seoul, Korea). HEK293T cells were purchased from ATCC (ATCC; Manassas, VA, USA). A549 and NCI-H460 cells were grown in RPMI 1640 (Corning Cellgro; Manassas, VA, USA) and HEK293T cells were grown in DMEM (Corning Cellgro), supplemented with 10% FBS in the presence of penicillin and streptomycin (Corning Cellgro; Manassas, VA, USA) in a humidified 5% CO_2_ incubator at 37°C. For the administration of TGF-β, cells were seeded at 2.5×10^4^ cells/ml, and 16 hours later, the cells were treated with 2.5 ng/ml of TGF-β for 48 hours. TGF-β, cycloheximide, and all other chemical reagents were purchased from Sigma-Aldrich (St. Louis, MO, USA).

### Immunoblot analysis

Cells were lysed in lysis buffer [0.5% Triton X-100, 20 mM Tris, pH 7.5, 2 mM MgCl_2_, 1 mM dithiothreitol (DTT), 1 mM EGTA, 50 mM β -glycerophosphate, 25 mM NaF, 1 mM Na_3_VO_4_, 100 mg/ml PMSF, and a protease inhibitor cocktail (Roche; Indianapolis, IN, USA)]. After adjustment of the protein concentration, proteins were resolved by SDS-PAGE and subjected to immunoblot analysis with the appropriate antibodies as follows: β-catenin (Santa Cruz Biotechnology, sc-7963 and sc-7199), PLK1 (Millipore, 05-844); phospho-PLK1^T210^ (Cell Signaling, 5472); N-cadherin (Sigma, C3865); E-cadherin (Cell Signaling, 4065); SNAI1 (Santa Cruz Biotechnology, sc-271977); SNAI2 (Cell Signaling, 9585); TSG6 (R&D, MAB2104); TCF4 (Santa Cruz Biotechnology, sc-166699); laminin γ2 (Santa Cruz Biotechnology, sc-28330); CD44 (Santa Cruz Biotechnology, sc-9960); Smad2/3 (Cell Signaling, 8685); p-Smad2^S465/S467^ (Cell Signaling, 18338); p-Ser (Santa Cruz Biotechnology, sc-81514); c-Jun (Santa Cruz Biotechnology, sc-74543); c-Fos (Santa Cruz Biotechnology, sc-52); Ki67 (Abcam, ab16667); RFP (Life Technologies, R10367); GST (Santa Cruz Biotechnology, sc-57753); β-actin (Sigma, A5441); GAPDH (Santa Cruz Biotechnology, sc-47724); and IgG (Santa Cruz Biotechnology, sc-2027). Immune complexes were revealed using an Odyssey infrared imaging system (LI-COR Biosciences; Lincoln, NE, USA). Intensity values were determined using Photoshop software.

### Quantitative reverse transcription polymerase chain reaction (qRT-PCR)

Total RNA was extracted from cells at 48 h after exposure to TGF-β and quantified by Nanodrop (Thermo Fisher Scientific, Waltham, MA, USA). Next, cDNA was generated with a First Strand cDNA Synthesis Kit (Thermo Fisher Scientific). The synthesized cDNA was mixed with SYBR Green Master Mix (Bio-Rad Laboratories, Hercules, CA, USA) and gene-specific primers, and then qRT-PCR was performed using a CFX96 Real-Time PCR system (Bio-Rad Laboratories). The primer sequences used are shown in Table S2.

### Lentivirus-based plasmid preparation, virus production, and infection

For lentiviral expression of mouse PLK1 (gene ID no. 18817), we used pLVX-TRE3G-eRFP and pLVX-Tet3G vectors (Clontech #631351; Palo Alto, CA, USA). Wild-type, T210D, and W414F/V415A mutant PLK1 were as described previously 20, 34. The wild-type and various versions of PLK1 were subcloned into pLVX-TRE3G-eRFP and lentivirus expressing PLK1 was generated according to the manufacturer's guide and previous reports 20, 35. For viral infection, cells were treated with the viral particles from pLVX-Tet3G and pLVX-TRE3G-eRFP-PLK1 in the presence of 10 µg/ml polybrene and 10 mM HEPES. The infected cells were selected using 500 µg/ml G418 for 5 days and 2 µg/ml puromycin for 2 days. The expression of PLK1 was induced with 2 µg/ml of doxycycline.

### Lentivirus-based shRNA preparation

For the loss of function experiments, we prepared lentivirus-based shRNA transfer plasmids targeting human PLK1 (gene access no. NM_005030) at positions 245-265 (AGATTGTG CCTAAGTCTCTGC) (shPLK#1) or positions 1424-1444 (AGATCACCCTCCTTAAATATT) (shPLK1#2) and human CTNNB1 (gene access no. NM_007614) at positions 252-272 (CCATGGAACCAGACAGAAAAG) (shCTNNB1#1) or positions 2279-2299 (GCTTGGAATGAGACTGCTGAT) (shCTNNB1#2), and then the lentivirus was generated. The infected cells were selected using 2 µg/ml puromycin for 2 days.

### Immunoprecipitation assay

Cell lysates were incubated with normal IgG (Santa Cruz Biotechnology), anti-PLK1 (Millipore), anti-β-catenin (Santa Cruz Biotechnology), or anti-TCF4 (Santa Cruz Biotechnology) antibodies for 16 h at 4°C with end-over-end mixing. This was followed by incubation with protein A/G agarose (Santa Cruz Biotechnology) for 2 h at 4°C. Immunoprecipitants were separated from the supernatants by centrifugation and washed four times with lysis buffer. Proteins were resolved by SDS-PAGE and analyzed by immunoblot.

### Glutathione S-transferase (GST) pull-down assay

GST-tagged-β-catenin was expressed in *Escherichia coli* strain *BL21* and purified using glutathione-sepharose 4B beads (GE Healthcare Life Sciences) according to the manufacturer's instructions and as described in a previous report 36. GST-β-catenin bound to glutathione-sepharose 4B beads were incubated with the lysate of HEK 293T cells expressing wild-type, FA (W414F/V415A), N-terminus (a.a.1-305), and C-terminus (a.a. 306-603) of Flag-Plk1 for 2 h at 4°C. The resins were washed four times with cell lysis buffer and subjected to SDS-PAGE and immunoblotting with anti-Flag (Sigma) and anti-GST (Santa Cruz Technologies) antibodies.

### *In vitro* kinase assay

For the expression and purification of Plk1 kinase, GST-tagged Plk1 protein was purified using glutathione-sepharose 4B beads (GE Healthcare Life Sciences), as described in the manufacturer's instructions and a previous report 37. In the* in vitro* Plk1 kinase assay, purified active Plk1 kinase, GST-tagged β-catenin, and radioactive [γ-^32^P] ATP were used. The GST-TCTP protein was used as a positive control. The samples were resolved by SDS-PAGE and the phosphorylation was detected by autoradiography.

### In-gel digestion with trypsin and peptide extraction

A Plk1 kinase assay was performed with the purified active version of Plk1 (T210D), GST-tagged β-catenin, and cold 25 µM ATP. After the phosphorylated GST-β-catenin proteins were resolved by SDS-PAGE, the protein bands from the SDS-PAGE were in-gel digested with trypsin and extracted for LC-MS/MS analysis as described in a previous report 35.

### LC-MS/MS analysis

LC-MS/MS analysis was performed as described in a previous report 35. The tolerance of the peptide mass was set to 10 ppm. The MS/MS ion mass tolerance was 0.8 Da, allowance of missed cleavage was 2, and charge states (+2 and +3) were taken into account for data analysis. Only significant hits were defined by MASCOT probability analysis.

### Generation of phosphomimetic and non-phosphomimetic β-catenin mutants

Using plasmids containing GST-tagged β-catenin (gene ID 1499), the putative phosphorylation sites of β-catenin were converted to Ala or Asp using mutagenesis primers. Mutagenesis was performed using a QuickChange II Site-Directed Mutagenesis Kit (Promega; Madison, WI, USA) according to the manufacturer's protocol. The primer sequences used for converting to Ala or Asp are shown in Table S3.

### Transwell-based cell migration and inverted invasion assay

Transwell-based cell migration and invasion assays were performed as described previously 35. Briefly, cell migration assays were conducted using 24-well plates with 8-μm-pore Transwell chambers (Corning, NY, USA). The lower chamber was filled with culture medium containing 10% FBS. A549 cells were suspended at a density of 5×10^4^ cells/ml in RPMI 1640, without FBS, and added to the upper chamber. Three days after seeding, the cells on the bottom layer surface were stained with 0.05% crystal violet dye, and the intensity values were measured using an Odyssey infrared imaging system (LI-COR Biosciences). For the cell invasion assay, cells were seeded in the upper chamber filled with Matrigel (BD Biosciences, Erembodegem, Belgium). Fourteen days after seeding, the cells on the bottom layer surface were stained with 0.05% crystal violet dye, and after treatment with DMSO, the absorbance was measured at 590 nm using an M4 microplate reader (Molecular Devices, CA, USA).

### Wound-healing assay

A549 cells were seeded at 2×10^5^ cells/ml, and a wound was established by scratching one time with a 1-mm-thick pipette tip. Wounded monolayer images were collected and analyzed with an Eclipse Ti microscope (Nikon, Tokyo, Japan) at the times indicated.

### Colony formation assay

Cells (5×10^3^ cells/ml) were resuspended in 2 ml of medium with 10% FBS in 0.4% agar and overlaid onto the bottom agar layer composed of 10% FBS and 0.6% agar in 1.5 ml of medium in a 35-mm dish. The cells were incubated in a humidified 5% CO_2_ incubator at 37°C. After 4 weeks, the colonies formed in the agar were counted after staining with 0.05% crystal violet.

### Transcriptome profiling

RNA was extracted from the indicated cells expressing mock, wild-type, phosphomimetic (S311D), or non-phosphomimetic β-catenin (S311A). RNA purity and integrity were evaluated using an ND-1000 spectrophotometer (Nanodrop, Wilmington, DE, USA) and Agilent 2100 Bioanalyzer (Agilent Technologies, Palo Alto, CA, USA). The Affymetrix whole transcript expression array process was conducted according to the manufacturer's protocol (GeneChip Whole Transcript PLUS reagent kit). cDNA was synthesized using a GeneChip WT (Whole Transcript) Amplification kit as described by the manufacturer. The sense cDNA was then fragmented and biotin-labeled with terminal deoxynucleotidyl transferase using a GeneChip WT Terminal labeling kit. Approximately 5.5 μg of labeled DNA target was hybridized to the Affymetrix GeneChip Human 2.0 ST Array at 45°C for 16 hours. Hybridized arrays were washed and stained on a GeneChip Fluidics Station 450 and scanned on a GCS3000 Scanner (Affymetrix). Signal values were computed using Affymetrix GeneChip Command Console software.

### Microarray analysis

Raw data were extracted automatically using an Affymetrix data extraction protocol in the Affymetrix GeneChip Command Console software. After importing CEL files, the data were summarized and normalized with a robust multi-average (RMA) method implemented in the Affymetrix Expression Console software. We exported the results of the gene-level RMA analysis and performed a differentially expressed gene analysis. The analysis comparing the invasive wild-type, S311A, or S311D β-catenin with the invasive mock was carried out using fold changes. For transcriptome data, gene probes with significant fold changes (more than 1.5) were clustered. To develop a significant probe list, we performed a gene-enrichment and functional annotation analysis using gene ontology (http://geneontology.org/) and KEGG (http://kegg.jp). All statistical tests and visualizations of differentially expressed genes were conducted using R statistical language v. 3.1.2. (www.r-project.org).

### Animal studies for lung metastasis

Four-week-old male BALB/c nude mice (Orient Bio Inc., Seoul, Korea) were injected with 1 × 10^6^ A549 cells (in 100 μl PBS) expressing pLVX-TRE3G-eRFP-Tet3G-Mock, WT β-catenin, β-catenin^mtGSK3β^ (S33/S37/T41/S45A), S311D of β-catenin^mtGSK3β^, S311A of β-catenin^mtGSK3β^ via the tail vein. The mice received 1 mg/ml of doxycycline in their drinking water to induce β-catenin overexpression. Twelve weeks after the injection, all mice were sacrificed, and their lungs were separated and fixed in 4% paraformaldehyde for H&E staining and Ki67 tissue staining. All animal experiments were approved and managed by the guidelines of the Institutional Animal Care and Use Committee, Hanyang University (HY-IACUC-2017-0115A).

### Immunofluorescence

A549 cells grown on coverslips were fixed with 4% paraformaldehyde. Methanol was used for permeabilization. Cells were washed three times with 0.1% Triton X-100 in PBS (PBST), incubated overnight at 4°C in PBST and 3% bovine serum albumin (BSA), and then incubated with anti-RFP (Life Technologies, R10367) and anti-α-tubulin (Sigma-Aldrich, MABT205) antibodies. The cells were washed three times with PBST and then incubated with Cy3-conjugated anti-rabbit secondary antibodies (Jackson ImmunoResearch Laboratories, West Grove, PA, USA), FITC-conjugated anti-mouse secondary antibodies (Jackson ImmunoResearch Laboratories), and 4′, 6-diamidine-2-phenylindole (DAPI) (Sigma-Aldrich) for DNA staining. Images of cells were collected and evaluated with a confocal microscope FW3000 (Olympus; Tokyo, Japan).

### Chromatin immunoprecipitation assays

A ChIP assay was performed as described 35. We examined the interaction between TCF4 and the promoters of *LAMC2*, *CD44*,* TNFAIP6*, and *JUN* in A549 cells expressing wild-type, S311A, or S311D β-catenin. Cross-linking was done with 1.4% formaldehyde. Cells were lysed with IP buffer. Chromatin was sheared by sonication and incubated with polyclonal antibodies to TCF4 (Santa Cruz Biotechnology, sc-166699) or normal IgG for 16 hours. Sheared chromatin was incubated with protein A/G beads (Santa Cruz Biotechnology) for 2 hours and washed five times with IP buffer. Chelex 100 slurry (Bio-Rad Laboratories) was added to the washed beads, which were then boiled and incubated with Proteinase K (Invitrogen; Carlsbad, CA, USA) at 55°C for 30 min. The samples were boiled again and cleared by centrifugation, and then the supernatants were taken for real-time PCR. The bound chromatin fraction was amplified with human of *LAMC2*, *CD44*,* TNFAIP6*, and *JUN* promoter-specific primers (Table S4) for 40 cycles. Real-time PCR was carried out on a CFX96 Real-Time PCR system (Bio-Rad Laboratories) using SYBR Green Master Mix (Bio-Rad Laboratories, #1708880). We also tested the interaction between c-Jun (AP-1) and the promoters of PLK1 in A549 cells expressing wild-type, S311A, or S311D β-catenin using specific anti-c-Jun antibody (Santa Cruz Biotechnology, sc-166699). The data were analyzed by the comparative C_T_(ΔΔC_T_) method.

### Statistical analysis

All data are given as means ± SDs of at least three independent experiments, each performed in triplicate. Results were analyzed for statistically significant differences using the Student's *t*-test or two-way ANOVA test, and statistical significance was set at *p* < 0.05. (*p < 0.05; **p < 0.01; ***p < 0.001; ^#^*p <* 0.05; ^##^*p <* 0.01; ^###^*p <* 0.001).

## Results

### Clinical relevance of PLK1 and β-catenin in metastatic NSCLC

**Figure 1:**
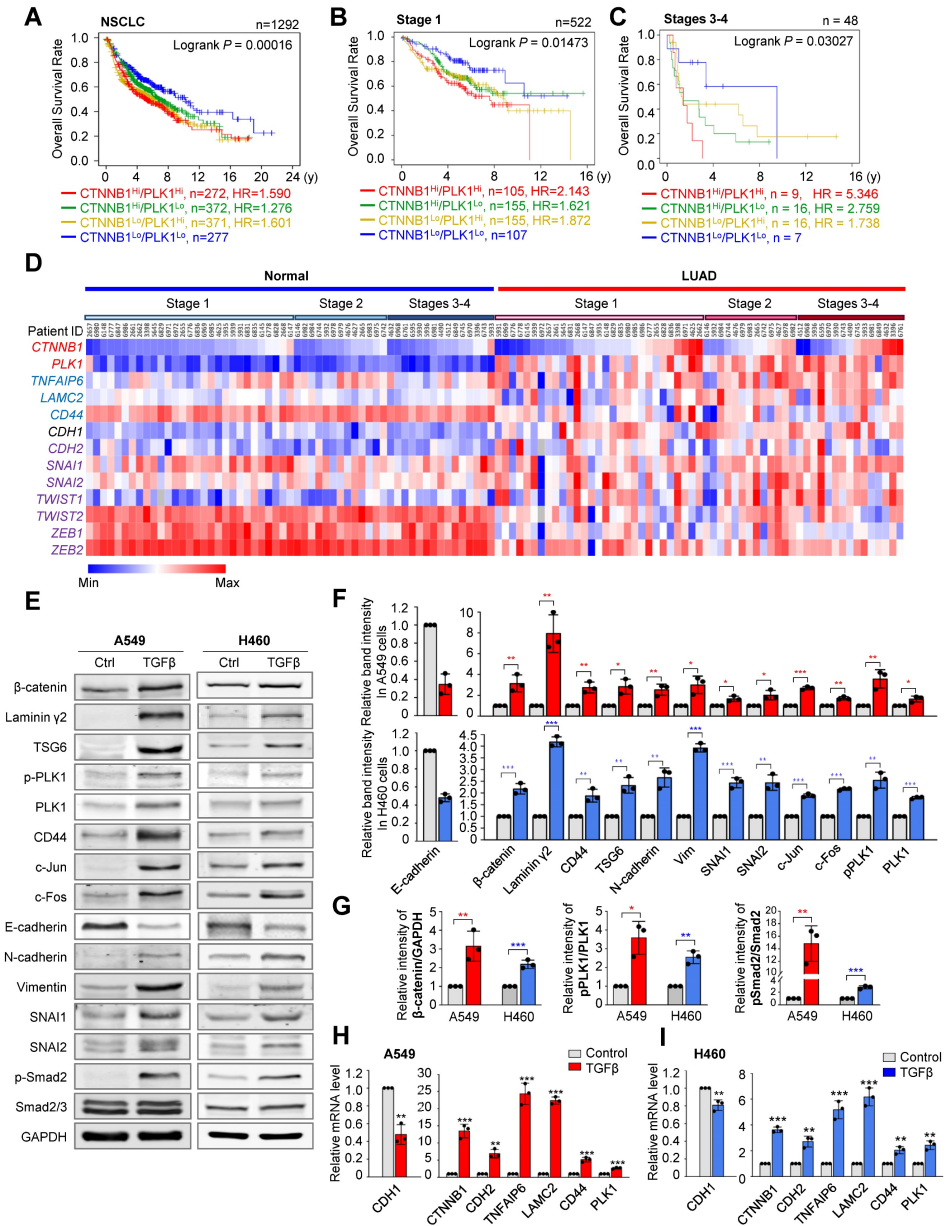
** Clinical relevance of PLK1 and β-catenin in metastatic NSCLC. A-C,** The overall survival (OS) times in NSCLC whole patients (*n*=1292) (**A**), NSCLC patients with stage 1 (*n*=522) (**B**), and stages 3-4 (*n*=48) (**C**) were analyzed according to their *PLK1* and *CTNNB1* expression levels using KM PLOTTER. High (Hi) and low (Lo) were generated by dividing patients according to their expression at the median cut-off. **D,** A heatmap analysis was performed from the TCGA dataset of lung adenocarcinoma patients for *CTNNB1, PLK1*, *TNFAIP6, LAMC2, CD44*, epithelial marker (*CDH1*), and mesenchymal markers (*CDH2, SNAI1, SNAI2, TWIST1, TWIST2, ZEB1*, and *ZEB2*) in paired normal (left) and tumor tissues (right) depending on stages. **E-F,** A549 and NCI-H460 (H460) non-small cell lung cancer cells were treated with 2.5 ng/ml of TGF-β for 48 hours. **E,** Immunoblotting was performed to measure the protein levels of β-catenin, laminin γ2, TSG6, CD44, c-Jun, c-Fos, PLK1, p-PLK1^T210^, p-Smad2^S465/S467^, Smad2/3, E-cadherin, N-cadherin, vimentin, SNAI1, SNAI2, and GAPDH in A549 (left panel) and NCI-H460 (H460) cells (right panel).** F**, The relative band intensities for β-catenin, laminin γ2, CD44, TSG6, c-Jun, c-Fos, PLK1, p-PLK1^T210^, E-cadherin, N-cadherin, vimentin, SNAI1, and SNAI2 were quantified using densitometry of Photoshop software. **G**, The relative band intensities for β-catenin/GAPDH, p-PLK1^T210^/PLK1, and p-Smad2^S465/S467^/Smad2 were quantified using densitometry of Photoshop software. **H-I,** qRT-PCR was performed for *CTNNB1*, *CDH1, CDH2*, *PLK1*, *TNFAIP6, LAMC2*, and* CD44* expression in A549 (**H**) and NCI-H460 (**I**) cells. Data are presented as mean ± SD of at least three independent experiments (significantly different as compared with experimental control). *p <0.05; **p <0.01; ***p <0.001.

In catalytically active PLK1-driven metastasis, *TNFAIP6* and *LAMC2* were within the top upregulated genes in microarray analysis 20 (Figure S1A). Hyaluronate and CD44, interacting proteins of TSG6 encoded by *TNFAIP6* and ECM adhesion factors 29, were upregulated in active PLK1-driven EMT (Figure S1B). When transcription factors were extracted from the Appyter Database of ChIP-Seq 38 using a list of the top 30 highly expressed genes in invasive cells expressing constitutively active PLK1 (T210D; TD), *TCF4* ranked within top three (Figure S1C). *CLOCK* and *TP53* were ruled out because of their basic function for circadian regulation and tumor suppression, respectively. Since *LAMC2* and *CD44* are the transcriptional target genes of β-catenin 24, 28, a binding partner of TCF4, for transcriptional regulation, we hypothesized that β-catenin can regulate active PLK1-driven metastasis as a transcription factor for expressing *LAMC2* and *CD44.* For this reason, the relationship between PLK1 and β-catenin, and their functions for regulating metastasis were investigated. First, to understand the clinical relevance between PLK1 and β-catenin in NSCLC, the overall survival (OS) of 1,292 NSCLC patients was analyzed using datasets (GSE14814, GSE29013, GSE31210, GSE37745, GSE50081, and GSE4573) from KM PLOTTER 33 (Figure 1A, Figure S2, and Table S1). OS of NSCLC patients with high *CTNNB1^Hi^/PLK1^Hi^* expression were significantly shorter than those with low *CTNNB1^Lo^/PLK1^Lo^* expression (n=272, HR=1.590) (Figure 1A, Table S1). The HR of patients having CTNNB1^Lo^/PLK1^Hi^ (yellow line, HR=1.601) is higher than that of patients having CTNNB1^Hi^/PLK1^Lo^ (green line, HR=1.276) in NSCLC patients (Figure 1A, Table S1). The OS patterns are more similar to those of lung adenocarcinoma (LUAD) patients than to those of lung squamous cell carcinoma (LUSQ) patients (Figure S2A-B, Table S1). Then we evaluated their clinical importance in primary and metastatic NSCLC. In 522 patients with stage 1 NSCLC, the OS of those with high *CTNNB1^Hi^/PLK1^Hi^* expression (n=105, HR=2.143) was shorter than those with low *CTNNB1^Lo^/PLK1^Lo^* expression (Figure 1B, Table S1). In a clinical analysis of 48 patients with stages 3 and 4 NSCLC, the OS of patients with high *CTNNB1^Hi^/PLK1^Hi^* expression (n=9, HR=5.346) were much shorter than those with low *CTNNB1^Lo^/PLK1^Lo^* expression (Figure 1C, Table S1). Therefore, the high expression of* CTNNB1/PLK1* was inversely correlated with the survival rates of NSCLC patients, especially in those with metastatic NSCLC. In addition, the HR of patients having *CTNNB1^Hi^/PLK1^Lo^* (green line, HR=2.759) was higher than that of patients having *CTNNB1^Lo^/PLK1^Hi^* (yellow line, HR=1.738) in stages 3 and 4 (Figure 1C). The HR in *CTNNB1^Hi^/PLK1^Lo^* patients with stages 3 and 4 (HR=2.759) is much higher than that of stage 1 (HR=1.621) (Figure 1B-C), indicating that the expression of *CTNNB1* would be inversely more dependent on the OS of patients in stages 3 and 4 than that of stage 1.

### Upregulation of β-catenin, TSG6, laminin γ2, and CD44 in TGF-β-induced or active PLK1-driven EMT

To observe the correlation of expression among *CTNNB1, PLK1,* and mesenchymal markers in LUAD depending on stage, The Cancer Genome Atlas (TCGA) data was used. Mesenchymal markers *SNAI1* and *ZEB2* were upregulated in stages 3-4. The expression levels and frequencies of PLK1 were higher in stages 3-4 than in those of stage 1 (Figure 1D). In the genomic analysis of LUAD, the levels of *CTNNB1* were higher in patients with metastatic stages 3 and 4 (46%, 7 tumors/15 total) than in those with tumor stage 1 (27%; 8 tumors/29 total) (Figure 1D), indicating that β-catenin and PLK1 are markedly expressed in metastatic NSCLC, especially in LUAD.

The changes in the levels of β-catenin protein and mRNA were observed during EMT induced by TGF-β in A549 and NCI-H460 cells (Figure 1E-I). In A549 and NCI-H460 cells treated with TGF-β, the expressions of mesenchymal markers N-cadherin (*CDH2*), vimentin (*VIM*), SNAI1 (*SNAI1*), and SNAI2 (*SNAI2*) were increased, and the epithelial marker E-cadherin (*CDH1*) was decreased (Figure 1E-F), consistent with the previous studies 20, 35. Under these conditions, the levels of the β-catenin protein and mRNA were upregulated, which also upregulated its target genes such as laminin γ2, CD44, and c-Jun (Figure 1E-I). The p-PLK1 was increased when p-Smad2 was upregulated in TGF-β-treated A549 and NCI-H460 cells (Figure 1G, middle panel). The relative levels of β-catenin/GAPDH and pPLK1/PLK1 were higher than those of the control in TGF-β-induced EMT (Figure 1G).

**Figure 2:**
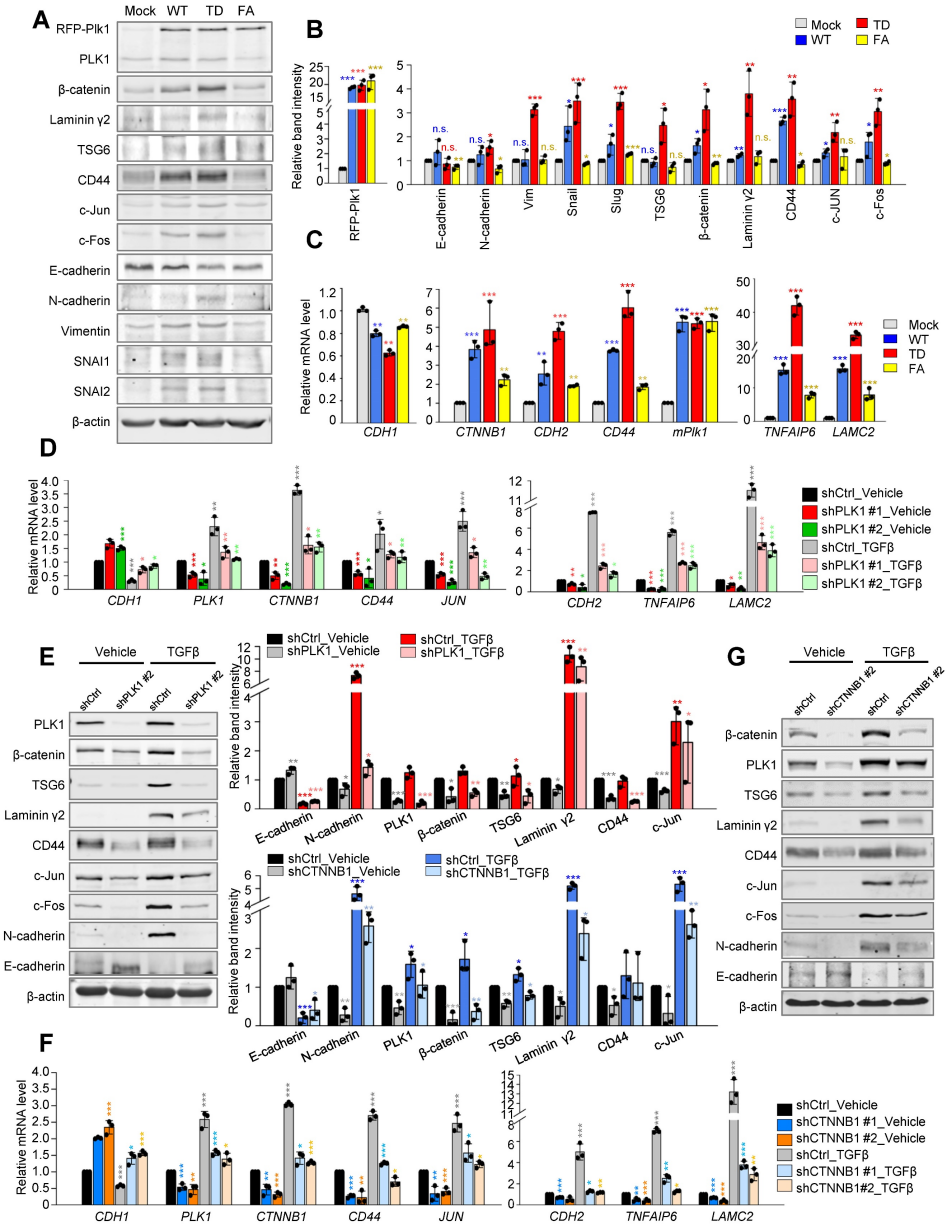
** Upregulation of TSG6, laminin γ2, and CD44 in active PLK1-driven EMT was disrupted by depletion of PLK1 or β-catenin. A-C,** The A549 cell expressing RFP-tagged WT, T210D (TD), and FA of PLK1 was expressed for 48 hours using a doxycycline-inducible system. A549 cells were treated with doxycycline to express RFP-tagged PLK1. **A,** Immunoblotting was performed using specific antibodies for PLK1, β-catenin, laminin γ2, CD44, TSG6, c-Jun, c-Fos, E-cadherin, N-cadherin, vimentin, SNAI1, SNAI2, and β-actin. **B,** The band intensity values were quantified using densitometry of Photoshop software, normalized, and plotted. **C,** qRT-PCR was performed for *CTNNB1*, *CDH1, CDH2*, *PLK1*, *TNFAIP6,* and* LAMC2* in A549 cells expressing wild-type or mutant PLK1. *p <0.05; **p <0.01; ***p <0.001; (*n*=3). Data are presented as mean ± SD. D-E, Cells with depleted PLK1 using specific shRNA#2 targeting human *PLK1* at positions 1424-1444, were treated with TGF-β for 48 hours. **D,** qRT-PCR was performed for *CDH1, CDH2*, *PLK1*, *TNFAIP6, CTNNB1, LAMC2, CD44*, and* JUN* expression in A549 cells with depleted PLK1. *p <0.05; **p <0.01; ***p <0.001; (*n*=3). Data are presented as mean ± SD. E, Immunoblotting was performed using specific antibodies for PLK1, β-catenin, TSG6, laminin γ2, CD44, c-Jun, c-Fos, N-cadherin, E-cadherin, and β-actin. The band intensity values were using densitometry of Photoshop software, normalized, and plotted (right upper panel). *p <0.05; **p <0.01; ***p <0.001; (*n*=3). Data are presented as mean ± SD. **F-G,** β-catenin-depleted cells using specific shRNA targeting *CTNNB1*, were treated with TGF-β for 48 hours. **F,** qRT-PCR was performed for *CDH1, CDH2*, *PLK1*, *TNFAIP6, CTNNB1, LAMC2, CD44*, and* JUN* expression in A549 cells with depleted β-catenin. **G,** Immunoblotting was performed to detect the levels of β-catenin, PLK1, TSG6, laminin γ2, CD44, c-Jun, c-Fos, N-cadherin, E-cadherin, and β-actin. The band intensity values were quantified using using densitometry of Photoshop software, normalized, and plotted (left lower panel). *p <0.05; **p <0.01; ***p <0.001; (*n*=3). Data are presented as mean ± SD.

Changes in the expression of β-catenin and its transcriptional targets were investigated in cells expressing the wild-type, constitutively active form (T210D; TD), and polo-box domain mutant (W414F/V415A; FA) of PLK1 20. In cells expressing TD-PLK1, levels of mesenchymal markers N-cadherin (*CDH2*), SNAI1 (*SNAI1*), and SNAI2 (*SNAI2*) were increased compared with those in cells expressing mock or FA (Figure 2A-B). Under these conditions, the levels of β-catenin, laminin γ2, CD44, and c-Jun increased in accordance with the expression of PLK1, and their expression was more upregulated in cells expressing active PLK1 (Figure 2A-C). In addition, the expression of *CTNNB1, LAMC2*, and *CD44* was also upregulated in cells expressing active PLK1 (Figure 2C). These data suggest that active PLK1 may regulate β-catenin during TGF-β or active PLK1-induced EMT.

To understand the correlation between β-catenin and PLK1, loss-of-function experiments were performed with shRNA targeting human PLK1 or β-catenin (Figure 2D-G, Figure S3). Expression of PLK1 was blocked in A549 cells using a lentiviral shRNA targeting PLK1 with/without TGF-β (Figure 2D, Figure S3A-C). Depletion of PLK1 using shRNA #1 or #2, reduced the levels of β-catenin protein regardless of the presence of TGF-β (Figure 2D, Figure S3B-C). At the same time, protein levels of TSG6, laminin γ2, CD44, and c-Jun proteins were downregulated in PLK1-depleted cells (Figure 2E, Figure S3B-C). Thus, β-catenin and its downstream factors including TSG6, laminin γ2, and CD44 could be affected by the presence of PLK1. Reciprocally, β-catenin was depleted in TGF-β-treated A549 cells (Figure 2F-G, Figure S3D-F). Knockdown of β-catenin using shRNA #1 or #2, downregulated the expression of N-cadherin, TSG6, laminin γ2, CD44, and c-Jun proteins (Figure 2G). When TGF-β was added to β-catenin-depleted cells, the levels of TSG6, laminin γ2, and CD44 were still downregulated whether TGF-β is present or not, indicating that the presence of β-catenin is important to their expression. Knockdown of β-catenin induced the downregulation of PLK1, which was partially recovered by TGF-β-induced EMT even when β-catenin is depleted (Figure 2G), indicating that PLK1 may partially be affected by β-catenin signaling during TGF-β-induced EMT. Depletion of PLK1 or β-catenin downregulates the expression of EMT factors including N-cadherin, vimentin, SNAI2, and p-Smad2 in a time-dependent manner even in TGF-β-induced EMT (Figure S3G-H). Taken together, the levels of β-catenin and its downstream factors including TSG6, laminin γ2, CD44, and c-Jun proteins are regulated by the presence and activity of PLK1, even in EMT.

### Phosphorylation of β-catenin at Ser311 through direct interaction with the polo-box domain of PLK1 in TGF-β-induced EMT during NSCLC

**Figure 3:**
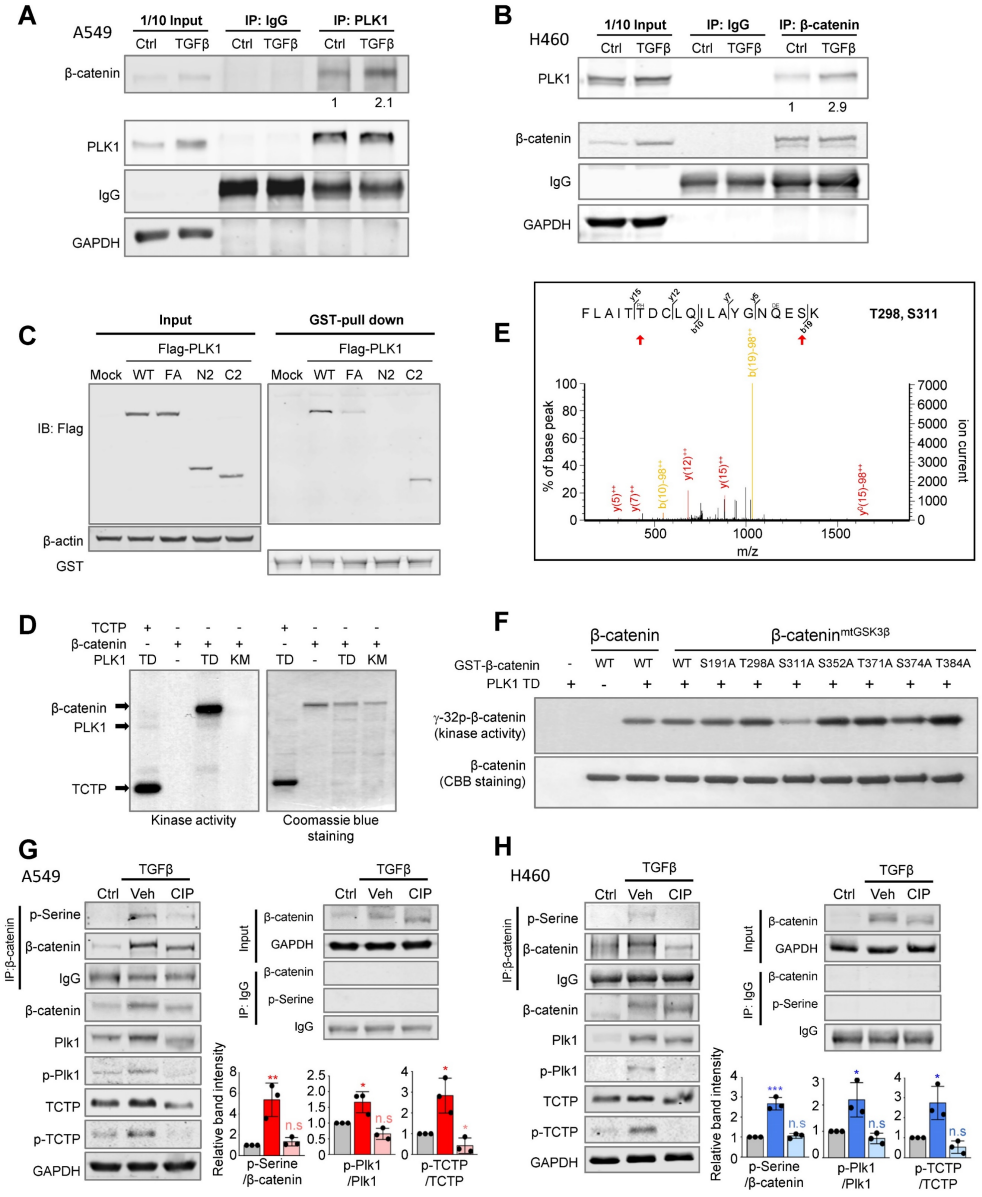
** Phosphorylation of β-catenin at Ser311 through direct interaction with the polo-box domain of PLK1 in TGF-β-induced EMT in NSCLC. A-B,** A549 **(A)** and NCI-H460 **(B)** were treated with TGF-β (2.5 ng/ml) for 48 hours. Immunoprecipitation of cell lysates was performed with normal IgG, anti-PLK1, or anti-β-catenin antibodies and then immunoblotting was done with anti-PLK1, anti-β-catenin, or anti-GAPDH antibodies. GAPDH was used as a loading control of immunoblotting in the input panel. **C,** A GST pull-down assay was performed. Purified GST-tagged β-catenin was incubated with lysates of HEK293T cells expressing Flag-tagged WT, FA (W414F/V415A), N-terminus (a.a.1-305), and C-terminus (a.a. 306-603) of PLK1. Immunoblotting was done with anti-Flag or anti-GST antibodies. **D,** An *in vitro* kinase assay was performed with an active version of PLK1 with T210D (PLK1-TD), inactive PLK1 (KM; K82M), radioactive ATP, and purified GST-tagged β-catenin. TCTP was used as the positive control as a PLK1 substrate. E, In the LC-MS/MS analysis, possible phosphorylation residues of β-catenin by PLK1 were newly detected at the Thr298 and Ser311 sites. **F,** Purified GST-tagged wild-type, S191A, T298A, S311A, S352A, T371A, S374A, and T384A mutants of β-catenin were used for a PLK1 kinase assay with radioactive ATP. **G-H,** Phosphorylation of β-catenin in A549 (G) and NCI-H460 (H) cells treated with TGF-β for 48 hours. Treatment with phosphatase (CIP) reduced the phosphorylation of β-catenin and PLK1 in TGF-β-induced EMT. Immunoprecipitation was performed with anti-normal or anti-β-catenin antibody and then immunoblotting was done with anti-p-Serine antibody. Immunoblotting was performed for β-catenin, PLK1, p-PLK1^T210^, TCTP, and p-TCTP^S46^ was done using specific antibodies. Data are presented as mean ± SD of at least three independent experiments (significantly different compared with experimental control). *p <0.05; **p <0.01; ***p <0.001.

We wanted to investigate how PLK1 affects the pathophysiology of β-catenin function. The interactome extracted from the GeneMANIA database 39 was analyzed and the physical interaction between PLK1 and β-catenin was displayed (Figure S4) based on their interaction in mitosis 15. However, their interaction and function during EMT are not clear. To investigate this, their interaction was examined by immunoprecipitation in TGF-β-induced EMT (Figure 3A-B). Immunoprecipitation in A549 or NCI-H460 cells during TGF-β-induced EMT, revealed that PLK1 interacts with β-catenin regardless of EMT induction, although the binding levels between PLK1 and β-catenin were much higher over two times in TGF-β-induced EMT cells compared with vehicle-treated control cells (Figure 3A-B). To examine the interacting domain of PLK1, GST pull-down assay was performed in HEK293T cells with GST-tagged β-catenin and various versions of Flag-tagged PLK1, including wild-type, mutant in polo-box-domain (W414F/V415A; FA), N-terminus having an ATP binding site, and C-terminus having polo-box domain (Figure 3C). The wild-type and C-terminus of PLK1 were bound with GST-β-catenin, but the N-terminus of PLK1 was not, indicating that the intact polo-box-domain of PLK1 is important in binding with β-catenin.

To analyze whether PLK1 phosphorylates β-catenin as a substrate in EMT, *in vitro* PLK1 kinase assay was performed (Figure 3D). β-catenin was phosphorylated by active PLK1 (TD) but not by inactive PLK1 (KM), and TCTP was used as a positive control. LC-MS/MS spectrometry analysis was performed to identify the phosphorylation sites of β-catenin. The analysis predicted that the sites in β-catenin that were phosphorylated by PLK1 were Ser191, Thr298, Ser311, Ser352, Thr371, Ser374, and Thr384 (Figure 3E; Figure S5A). Site-directed mutagenesis was performed for the replacement of these predicted residues with alanine. To rule out GSK3β and CK1α-mediated phosphorylation and degradation of β-catenin, the substituents with alanine residues at the phosphorylation sites of β-catenin (β-catenin^mtGSK3β^, S33A/S37A/T41A/S45A) by GSK3β (Ser33, Ser37, and Thr41) and by CK1α (Ser45) were used (Figure S5B-C). *In vitro* kinase assay revealed that the Ser311 in β-catenin is the site phosphorylated by PLK1 (Figure 3F). This residue is evolutionarily conserved in several species (Figure S5D).

To investigate whether active PLK1 phosphorylates β-catenin in EMT, phosphatase was treated in A549 and NCI-H460 cells after treatment with TGF-β for inducing EMT (Figure 3G-H). Phosphatase treatment reduced the levels of p-β-catenin^Ser^, p-PLK1^T210^, and p-TCTP^S46^ and delayed the shifted bands of total PLK1, TCTP, and β-catenin. Thus, β-catenin interacts with the polo-box domain of PLK1 in TGF-β-induced EMT of NSCLC and PLK1 phosphorylates β-catenin at Ser311 through direct interaction.

### Phosphorylated β-catenin at Ser311 promotes cell motility and invasiveness of NSCLC, but Phosphorylated β-catenin at Ser60 does not

**Figure 4:**
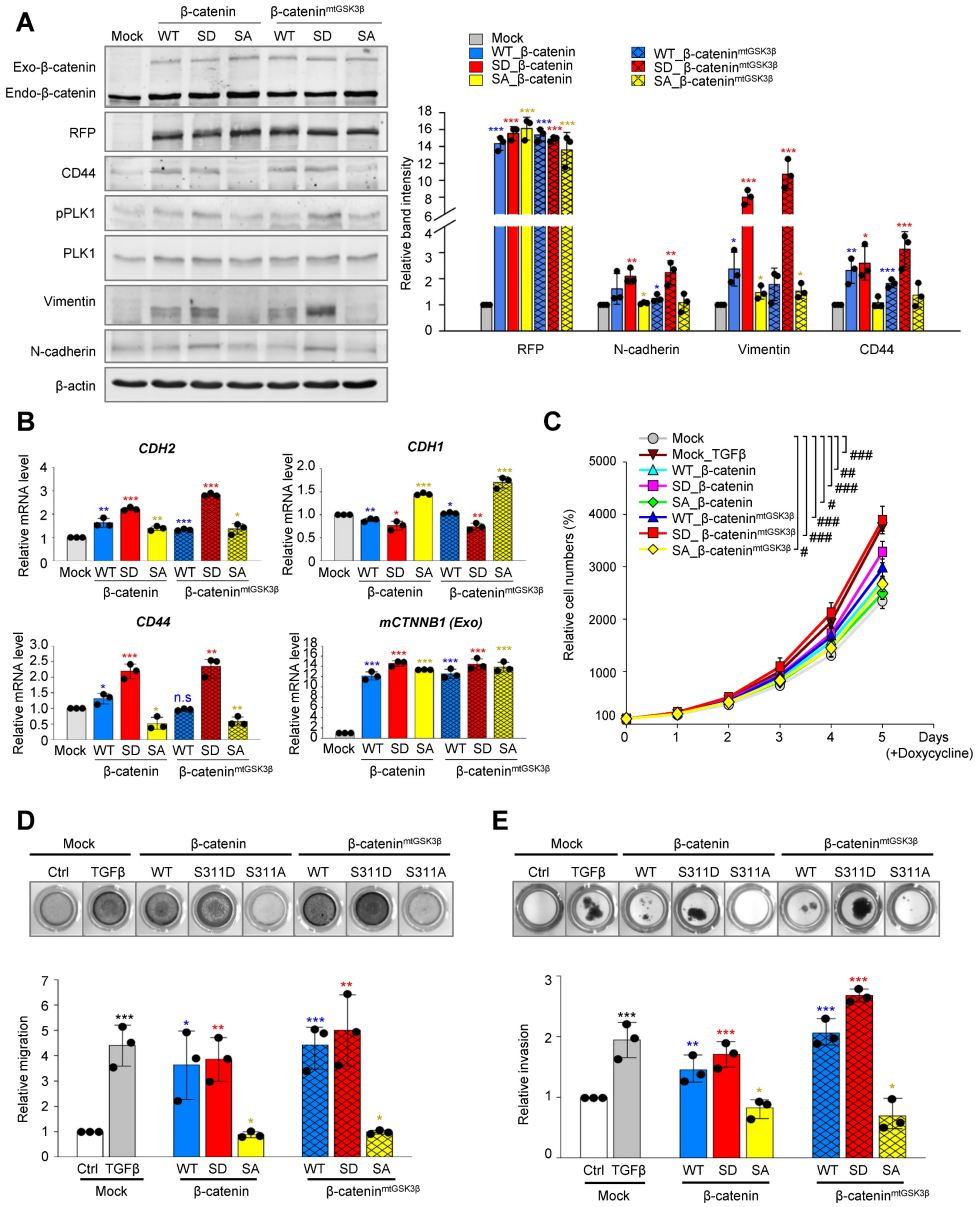
** Phosphorylated β-catenin promotes cell mobility and invasiveness of NSCLC. A-B**, RFP-tagged wild-type (WT), S311D, or S311A of β-catenin and WT at S311, S311D, S311A of β-catenin^mtGSK3β^ (S33/S37/T41/S45A) mutants were expressed in A549 cells. A549 cells were treated with doxycycline to express RFP-tagged β-catenin. **A,** Immunoblotting was performed using specific antibodies for β-catenin, RFP, CD44, PLK1, p-PLK1, vimentin, N-cadherin, and β-actin (left panel). The band intensity values were quantified using densitometry of Photoshop software, normalized, and plotted (right panel). **B,** qRT-PCR was performed for *CDH1*, *CDH2, CD44,* and *CTNNB1* in A549 cells expressing wild-type or mutant β-catenin. *p <0.05; **p <0.01; ***p <0.001; (*n*=3). Data are presented as mean ± SD. **C,** Cell proliferation assay was performed (*n*=3). Data are presented as mean ± SD of at least three independent experiments. #, *p* < 0.05; ##, *p* <0.01; ###, *p* <0.001 compared with indicated groups of cells. **D,** Cells expressing wild-type or mutants of β-catenin were subjected to a Transwell migration assay. As a positive control for migration, cells were treated with TGF-β. Three days after seeding, the cells on the bottom layer surface were stained with 0.05% crystal violet dye. Images of the Transwell cell migration assay were collected and analyzed with an Odyssey infrared imaging system (LI-COR Biosciences) and plotted. *p <0.05; **p <0.01; ***p <0.001 compared with experimental control. **E,** An invasion assay was performed using A549 cells expressing wild-type or mutants of β-catenin. Fourteen days after seeding, the cells that invaded the bottom layer surface were stained with 0.05% crystal violet dye, and the relative absorbance was plotted. Data are presented as mean ± SD of at least three independent experiments (significantly different compared with experimental control). *p <0.05; **p <0.01; ***p <0.001.

Based on the evidence that β-catenin modulates EMT 40, we wanted to investigate whether PLK1 phosphorylates β-catenin at Ser311, which can regulate EMT in NSCLC. For this, wild-type, phosphomimetic (S311D; SD), and non-phosphomimetic (S311A; SA) versions of β-catenin were expressed using the doxycycline-inducible system in NSCLC. Since β-catenin is unstable when Ser33, Ser37, Thr41, and Ser45 are phosphorylated by GSK3β and CK1α in cells in the absence of Wnt 8, 9, the sites were substituted by alanine for its stability in the version of β-catenin^mtGSK3β^ and the Ser311 residue by PLK1 was also substituted by alanine or aspartate (Figure 4A). With or without the substitution of alanine at phosphorylation sites by GSK3β (β-catenin^mtGSK3β^), cells expressing phosphomimetic β-catenin at S311 (S311D) upregulated the levels of CD44, p-PLK1, and mesenchymal markers including N-cadherin and vimentin (Figure 4A). The relative mRNA levels of *CDH2* and *CD44* were upregulated, whereas those of *CDH1* were downregulated in cells expressing the S311D of β-catenin (Figure 4B). A proliferation assay showed that the proliferation of cells expressing the S311D version of β-catenin^mtGSK3β^ was similar to that of cells treated with TGF-β (Figure 4C), which was slightly higher than that of cells expressing the S311D of β-catenin. Thus, the expression of phosphomimetic β-catenin at S311 induces mesenchymal factors for EMT.

Because of a previous study that β-catenin is phosphorylated at Ser60 by PLK1 during mitosis 14, S60D and S60A of β-catenin were also evaluated for their function in EMT using qRT-PCR and immunoblotting (Figure S6A-B). The mRNA and protein levels of E-cadherin, N-cadherin, SNAI1, SNAI2, and vimentin were not changed by expressing S60D or S60A β-catenin, while those of cells expressing wild-type were upregulated (Figure S6A-B). In addition, the relative cell migration levels were not different between cells expressing S60D and S60A β-catenin (Figure S6C). However, the proliferation of cells expressing S60D β-catenin increased compared with that of cells expressing S60A β-catenin (Figure S6D). Therefore, p-S60-β-catenin functions in cell proliferation, but not in EMT.

To examine whether phosphorylation of β-catenin at S311 induces cell migration and invasiveness in NSCLC, Transwell cell migration, wound healing, and invasion assays were performed (Figure 4D-E, Figure S7A-B). Transwell cell migration and wound healing assays using phosphomimetic or non-phosphomimetic β-catenin revealed that phosphomimetic β-catenin at S311 promotes cell migration in A549 cells (Figure 4D; Figure S7A-B). In addition, the relative migration was higher in cells expressing the S311D of β-catenin^mtGSK3β^ sthan in cells expressing the single mutant of the S311D of β-catenin (Figure 4D). Depletion of β-catenin using shRNA suppressed the migration of A549 cells induced by TGF-β treatment (Figure S7C-D). Furthermore, we performed an invasion assay using Matrigel to evaluate the invasiveness of cells expressing phosphomimetic β-catenin (Figure 4E). Higher invasiveness was found in proteins expressing phosphomimetics than in those expressing non-phosphomimetics, which is consistent with cell motility (Figure 4E). Cells expressing S311D β-catenin^mtGSK3β^ were more invasive than cells expressing S311D β-catenin, indicating that the stability of β-catenin can increase cell migration and invasion (Figure 4E).

Therefore, the phosphorylation of β-catenin at Ser311 by PLK1 promotes cell migration and invasiveness in NSCLC.

### Phosphorylation of β-catenin at S311 by PLK1 induces metastasis in an *in vivo* model

**Figure 5:**
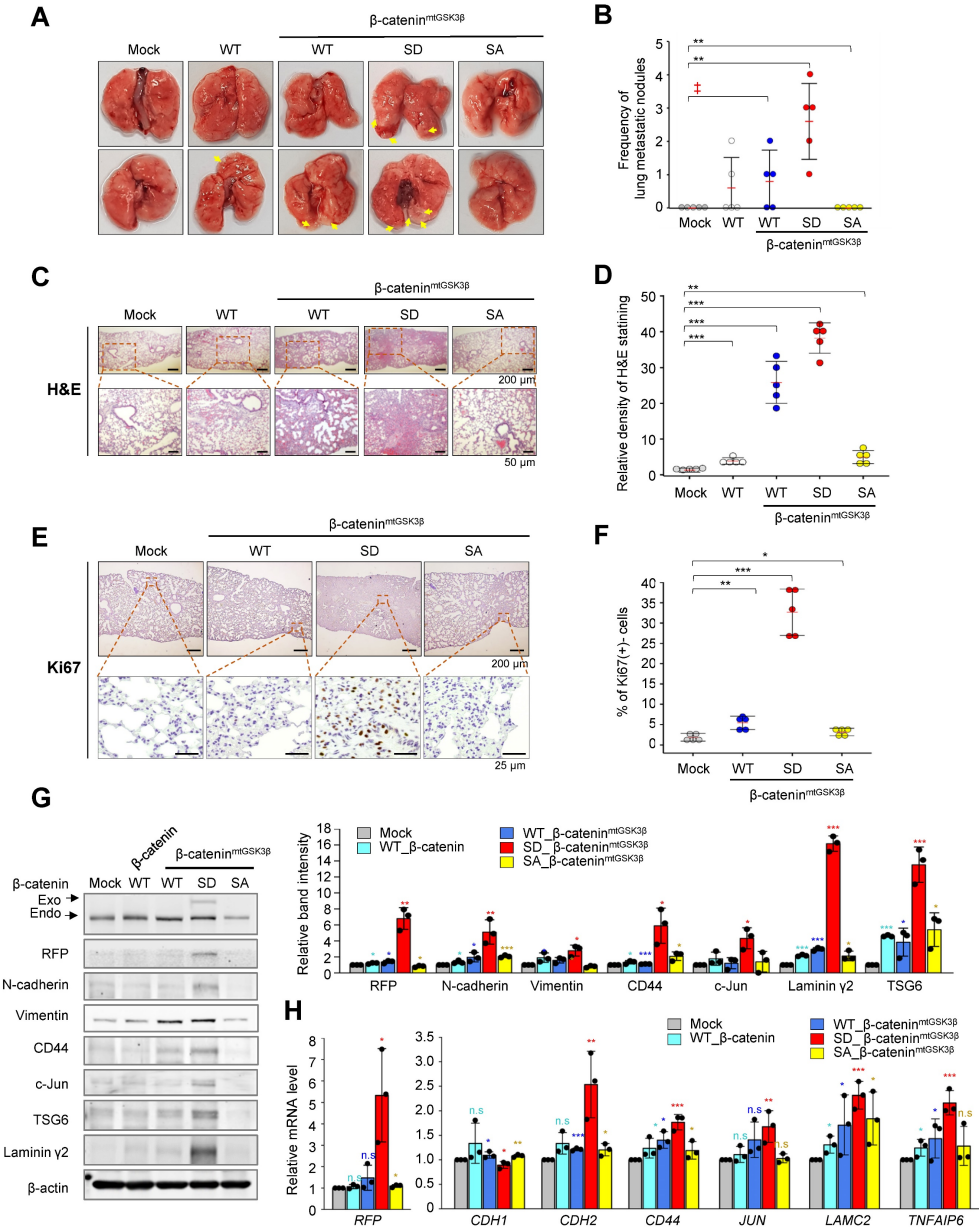
** Phosphorylated β-catenin at S311 by PLK1 induced metastasis in an *in vivo* model.** A549 cells expressing RFP-tagged WT of β-catenin and WT at S311, S311D, or S311A of β-catenin^mtGSK3β^ were injected intravenously into the tail veins of four-week-old BALB/c nude mice, and the tumorigenic and metastatic properties were evaluated after 15 weeks. **A,** Representative lung tumors from the mouse model. **B,** The number of metastatic lung tumors was counted and plotted (*n* = 5). Data are presented as mean ± SD. **C-F,** Representative H&E staining (**C-D**) and Ki-67 staining (**E-F**) were performed using lung tissue from the mice. The relative density of H&E staining (**D**) and Ki-67 staining (**F**) was analyzed and plotted. *p <0.05; **p <0.01; ***p <0.001. Data are presented as mean ± SD. **G,** Immunoblotting was performed using lung tissue lysates from each mouse model. β-catenin, RFP, N-cadherin, vimentin, CD44, c-Jun, TSG6, laminin γ2, and β-actin were detected using specific antibodies. The band intensity values were quantified using densitometry of Photoshop software, normalized, and plotted (left panel). *p <0.05; **p <0.01; ***p <0.001. **H,** qRT-PCR was performed for *RFP, CDH1, CDH2, CD44, JUN, LAMC2,* and *TNFAIP6* using lung tissue lysates from each mouse model. The relative mRNA expression was plotted. *p <0.05; **p <0.01; ***p <0.001.

The expression of β-catenin phosphomimetic at S311 promotes cell motility and invasiveness in NSCLC (Figure 4). To determine whether phosphorylation of β-catenin at S311 by PLK1 promotes metastasis *in vivo*, cells expressing β-catenin phosphomimetic at S311 were injected into the tail veins of BALB/c mice (n=5). After 10 weeks, the frequency of lung metastasis nodules was the greatest in the mice injected with cells expressing phosphomimetic β-catenin^mtGSK3β^ (S311D of β-catenin^mtGSK3β^) compared with those of other versions of β-catenin or β-catenin^mtGSK3β^ (Figure 5A-B). In the mice injected with cells expressing non-phosphomimetic β-catenin^mtGSK3β^ (S311A of β-catenin^mtGSK3β^), cancer metastasis and tumor formation were not detected (Figure 5A-B), indicating that phosphorylation of β-catenin at S311 promotes lung metastasis in NSCLC. H&E staining for tumor formation and Ki67 staining for the degree of proliferation demonstrated that cells expressing phosphomimetic β-catenin^mtGSK3β^ (S311D of β-catenin^mtGSK3β^) increased tumorigenesis and cell proliferation in metastatic nodules, while S311A in β-catenin^mtGSK3β^ did not affect tumorigenesis and cell proliferation (Figure 5C-F).

To clarify whether p-β-catenin^S311^ induces EMT and metastasis in the lung tissues of mice, the levels of N-cadherin, vimentin, and the transcriptional targets of β-catenin were observed (Figure 5G). RFP-tagged exogenous β-catenin was observed in mice injected cells expressing S311D β-catenin^mtGSK3β^ (Figure 5G), indicating that cells with S311D β-catenin^mtGSK3β^ more easily survived in the mouse model, which may be related with tumor formation. Cells with exogenous β-catenin were less observed in the lung tissues of the mice as tumors were formed with approximately 0~2 nodules. In addition, in mice injected with cells expressing S311D β-catenin^mtGSK3β^, mesenchymal markers including N-cadherin and vimentin were upregulated. Moreover, the expressions of CD44, c-Jun, and TSG6, transcriptional targets of β-catenin, increased in mice injected with cells expressing S311D β-catenin^mtGSK3β^ (Figure 5G). These patterns were similar to the mRNA levels of *RFP*, *CDH2*, *CD44*, *JUN*, and *TNFAIP6* (Figure 5H). Therefore, phosphorylation of β-catenin at Ser-311 by PLK1 promotes cancer metastasis *in vivo*.

### Phosphorylation of β-catenin at Ser311 facilitates its stability and translocalization into the nuclei in NSCLC

**Figure 6:**
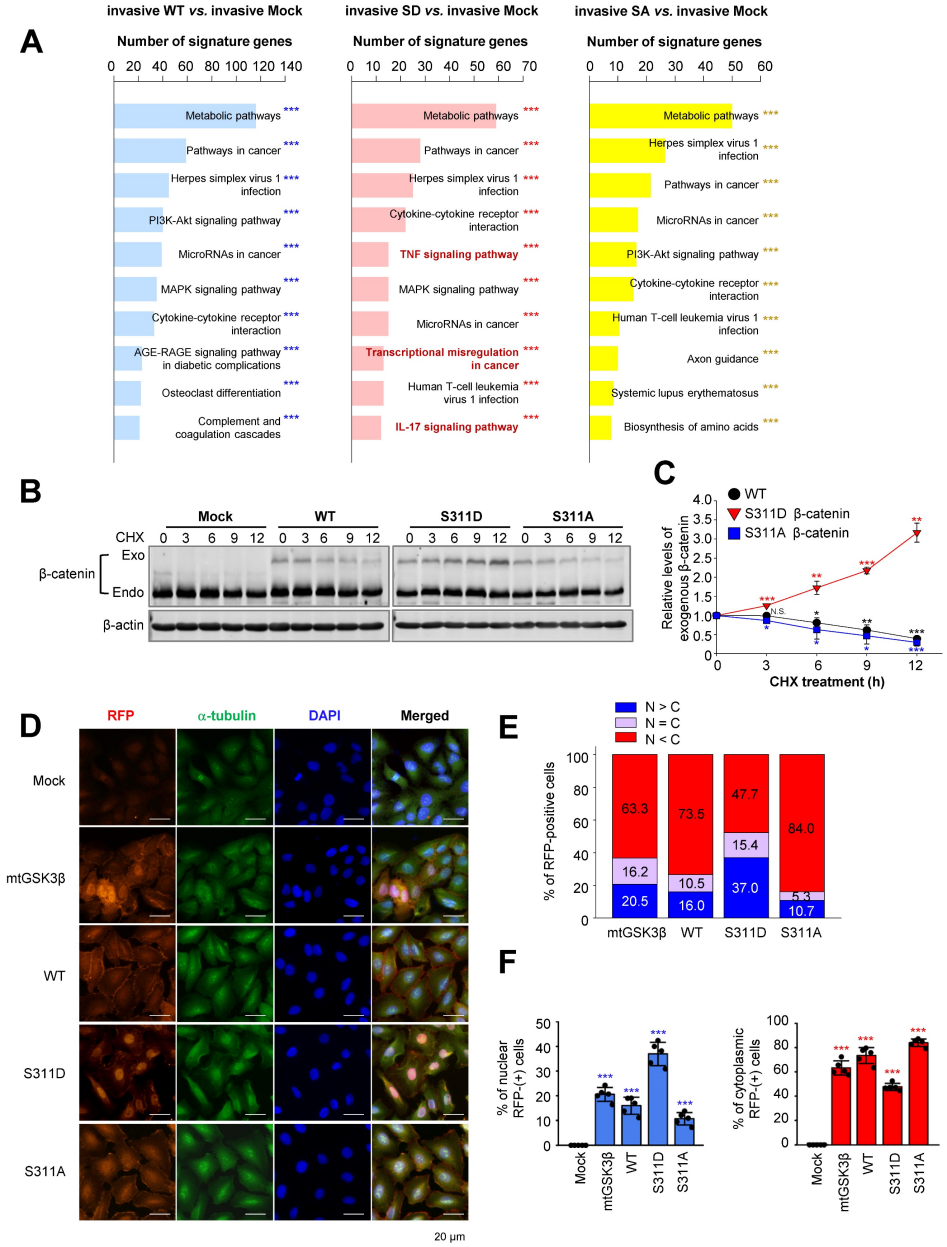
** Phosphorylation of β-catenin at Ser311 facilitates its stability and translocalization into the nuclei in NSCLC. A,** KEGG pathways functional analysis in invasive or non-invasive A549 cells expressing wild-type (WT), S311D (SD), or S311A (SA) of β-catenin. The invasive A549 cells expressing wild-type or mutants of β-catenin, transcriptome profiles were analyzed by microarray. The transcriptome data were clustered by gene probes and fold changes >1.5 indicated significant changes in cells expressing wild-type (WT), S311D (SD), or S311A (SA) of β-catenin. Gene Ontology (GO) and KEGG pathway analysis of transcriptome profiles was performed. The top 10 terms of KEGG pathways were analyzed, and the signaling pathways based on the number of signature genes in cells expressing invasive WT (left panel), SD (middle panel), or SA (right panel) compared to those expressing mock. ***p <0.001. **B-C,** A549 cells expressing RFP-tagged WT, S311D, or S311A of β-catenin were treated with 100 µg/ml cycloheximide in a time-dependent manner. **B,** Immunoblotting was performed to detect the levels of exogenous β-catenin using anti-β-catenin antibody. **C,** The relative levels of exogenous β-catenin were quantified using LI-COR Odyssey software (Li-COR Biosciences), normalized, and plotted. *p <0.05; **p <0.01; ***p <0.001. **D,** Immunofluorescence was performed with A549 cells expressing wild-type (WT), S311D β-catenin, S311A β-catenin, and β-catenin^mtGSK3β^ (mtGSK3β). RFP protein (red), α-tubulin (green), and DNA (DAPI, blue) are displayed. Scale bar, 20 µm. **E-F,** Quantification of the population of cells in the cytoplasm, nucleus, and both. n> 800.

Microarray analysis was performed to identify distinct pathways in an invasive single-point mutant at Ser311 of β-catenin with alanine or aspartate in A549 cells (Figure 6A). Among the top 10 terms in a KEGG pathway functional analysis in invasive cells expressing S311D β-catenin compared with invasive mock cells, pathways related to transcriptional misregulation in cancer were detected as distinguished pathways (Figure 6A, middle). Because β-catenin is known as a transcriptional factor for TSG6, laminin γ2, CD44, and c-Jun, and their levels of expression were regulated by the activity of PLK1 (Figure 2) and its transcriptional activity depended on its stability, we wanted to investigate whether the stability of β-catenin is regulated by PLK1-mediated phosphorylation. For this, the single-point mutant at Ser311 of β-catenin with alanine or aspartate was expressed in A549 cells (Figure 6B-C). The levels of mock, wild-type, phosphomimetic, and non-phosphomimetic β-catenin were observed after treatment with cycloheximide to block protein biosynthesis in a time-dependent manner. Over time, the levels of wild-type and non-phosphomimetic β-catenin became lower. However, the levels of phosphomimetic β-catenin increased by approximately 3.5-fold at 12 hours after treatment with cycloheximide (Figure 6B-C), indicating that the stability of β-catenin is regulated by phosphorylation at Ser311 by PLK1.

Because β-catenin is a transcriptional factor, we wanted to understand whether stabilized β-catenin is translocated into the nucleus for transcription. For this, an immunofluorescence assay was performed in A549 cells expressing mock, wild-type, S311D β-catenin, S311A β-catenin, and β-catenin^mtGSK3β^ (Ser33/Ser37/Thr41/Ser45A) (Figure 6D-F). The wild-type of β-catenin was mainly located in the cytoplasm in up to 73.5% of cells and in approximately 16% of cells in the nucleus (Figure 6D-F). The stable β-catenin^mtGSK3β^ was located in the cytoplasm and nucleus with a ratio of approximately 63% *vs.* 20%. The nuclear phosphomimetic S311D of β-catenin was higher by approximately 37% than that of wild-type, S311A β-catenin, and higher than the β-catenin^mtGSK3β^ (Ser33/Ser37/Thr41/Ser45A) (37% *vs.* 16%, 10.7%, or 20.5%), indicating that phosphorylation of β-catenin at S311 by PLK1 facilitated localization into the nucleus.

### Phosphorylation of β-catenin at Ser311 enhances the transcriptional activity that is connected with β-catenin/AP-1/PLK1 axis signaling

**Figure 7:**
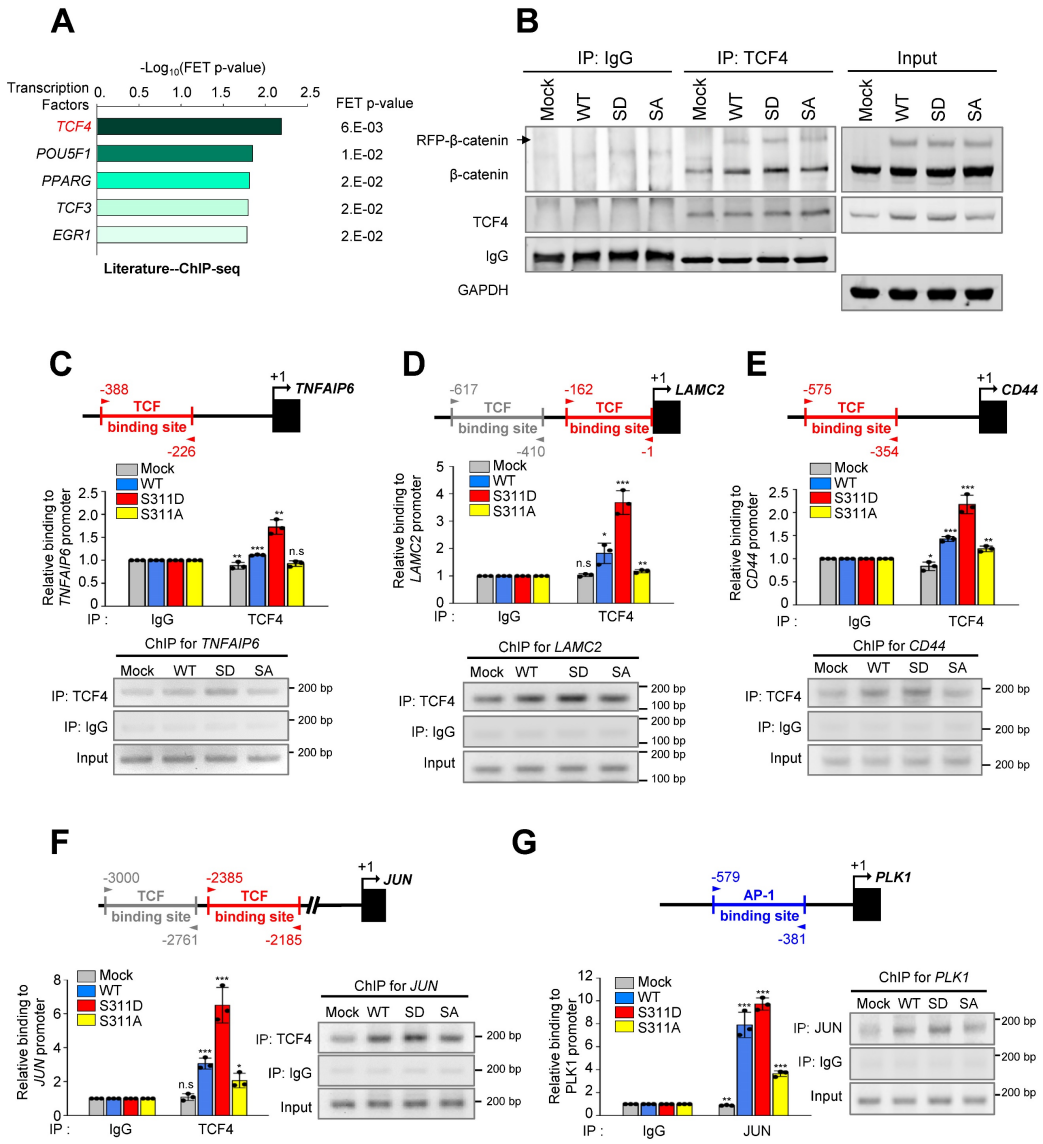
** Phosphorylation of β-catenin at Ser311 enhances its transcriptional activity of β-catenin/AP-1/PLK1 axis signaling. A,** Prediction of transcriptional factors of *TNFAIP6*, *LAMC2*, *CD44*, and *JUN* was performed, and the top five factors were extracted from APPYTER. **B,** A549 cells expressing RFP-tagged WT, S311D, or S311A of β-catenin were prepared for immunoprecipitation with anti-TCF4 antibody. Immunoblotting was performed with anti-β-catenin and TCF4 antibodies. **C-F**, ChIP assays for TCF4 binding to the promoters of *TNFAIP6* (C), *LAMC2* (D),* CD44* **(E)**, and *JUN* **(F)**. Assays were performed on chromatin fragments using TCF4 antibody and normalized to pre-immune normal IgG. Immunoprecipitated fractions were assayed by PCR for binding to the promoters of *TNFAIP6* (C), *LAMC2* (D),* CD44* (E), and *JUN* (F). **G,** ChIP assays for AP-1 binding to the promoters of *PLK1*. Immunoprecipitated fractions were assayed by PCR for binding to the promoters of *PLK1*. The PCR products were visualized in agarose gels. Data are presented as mean ± SD of three independent experiments (significantly different as compared with experimental control). *p <0.05; **p <0.01; ***p <0.001; (*n*=3).

We then investigated whether PLK1-mediated phosphorylation of β-catenin can increase transcriptional activity. In Figure 6A, transcriptional misregulation in cancer was detected as a distinguished pathway among the top 10 terms of a KEGG pathway functional analysis in invasive cells expressing SD compared with invasive mock cells. Analysis of transcription factors extracted from the Appyter Database of ChIP-Seq 38 revealed that TCF4 is a main factor for the expression of *TNFAIP6*, *LAMC2*, *CD44*, and *JUN* (Figure 7A). Since β-catenin binds with TCF4/LEF 41, the binding of phosphomimetic β-catenin with TCF4/LEF was assessed. For this, TCF4 was immune-precipitated using anti-TCF4 antibody and immunoblotting was performed with anti-β-catenin (Figure 7B). The results showed that TCF4 interacted with the phosphomimetic version of β-catenin more than the wild-type or non-phosphomimetic version, indicating that PLK1-mediated phosphorylation of β-catenin facilitates the interaction with TCF4/LEF. To understand the increase of laminin γ2, CD44, TSG6, or c-Jun/c-Fos in mice expressing S311D of β-catenin^mtGSK3β^ (Figure 5G), in A549 cells expressing PLK1-TD (Figure 2A), or in S311D of β-catenin/ β-catenin^mtGSK3β^ (Figure 4A), the TCF4/LEF binding motif was analyzed in their promoter regions (Figure S8A). TCF-binding elements are characterized by a highly conserved consensus sequence with 5′-CTTTG(A/T)(A/T)-3′ 41, 42. Based on the analysis, a ChIP assay was performed to observe whether the expression of these genes is achieved by S311D β-catenin complexed with TCF4/LEF after nuclear translocation. The TCF-binding elements are recognized at the promoter of *TNFAIP6* (Figure S8A). A ChIP assay was performed to observe whether *TNFAIP6* expression is activated by S311D β-catenin complexed with TCF4/LEF. *TNFAIP6* was upregulated weakly approximately 1.7-times by TCF4 binding to their promoters in cells expressing S311D β-catenin compared with that of the mock (Figure 7C). *LAMC2* was upregulated approximately 3-times by TCF4 binding to their promoters in cells expressing S311D β-catenin compared with that of the mock (Figure 7D). Whereas in cells expressing S311A β-catenin, the levels of *LAMC2* were similar to those of the mock. In the expression of *CD44* and *JUN*, the patterns were similar to that of *LAMC2* (Figure 7D-F). Thus, p-S311-β-catenin binds with TCF4 and upregulates the expression of *LAMC2*, *CD44*, *TNFAIP6*, and *JUN*.

Since c-Jun is a component of AP-1 and the PLK1 promoter regions have the AP-1 binding motif 30 (Figure S8B), a ChIP assay was performed with PLK1 using anti-c-Jun antibody to determine whether AP-1 can activate the expression of PLK1 in cells expressing S311D β-catenin. Of note, the expression of PLK1 was highly upregulated by approximately 7- and 9-times by c-Jun binding to its promoter in cells expressing WT and S311D β-catenin, respectively, compared with that of the mock (Figure 7G). However, the levels of PLK1 in cells expressing non-phosphomimetics were 3-times lower than the WT (Figure 7G), indicating that the expression of PLK1 by AP-1 is amplified by phosphorylated β-catenin through the direct expression of c-Jun.

## Discussion

Previously we observed that *TNFAIP6* and* LAMC2* are highly expressed within the top three genes in invasive PLK1-induced EMT 20. However, it is not clearly understood, which factors regulate these events. Prediction of transcriptional regulators for ECM factors using a ChIP-Seq database and studies on β-catenin in colorectal cancer 31, 43 provide the possibility that β-catenin functions as a transcriptional factor for EMT of NSCLC. Here, we have found that a central component of Wnt signaling and a transcriptional regulator in EMT, β-catenin 2, 40, 41, drives the expressions of *TNFAIP6*,* LAMC2*,* CD44*, and *JUN* during active PLK1-driven or TGF-β-induced EMT, functioning as a transcriptional factor in NSCLC.

More specifically, in TGF-β-induced EMT, the highly activated PLK1 interacts with and phosphorylates β-catenin at Ser311 through direct binding with the polo-box domain of PLK1 44. Because β-catenin can be unstable in the absence of Wnt by phosphorylation at Ser33, Ser37, and Ser45 by GSK3β and Thr41 by CK1α 8, 9, the sites were substituted by alanine for its stability in β-catenin^mtGSK3β^ and the Ser311 residue phosphorylated by PLK1 was also substituted by alanine or aspartate to investigate the effects on the expression of EMT markers, migration, and invasiveness (See Figure 4). Regardless of the alanine substitution at GSK3β and CK1α (β-catenin^mtGSK3β^) phosphorylation sites, cells expressing phosphomimetic β-catenin at Ser311 upregulated the levels of CD44, p-PLK1, and mesenchymal factors and promoted cell motility and invasiveness of NSCLC. Notably, phosphorylation of β-catenin at Ser311 increased its stability without the substitution of alanine at phosphorylation sites by GSK3β and CK1α (β-catenin^mtGSK3β^) (See Figure 6). These data indicate that the single phosphorylation at Ser311 would be important in maintaining the stability of β-catenin in the absence of Wnt. A single mutation at Ser311 to aspartate in β-catenin promotes its stability and translocalization into the nucleus for transcriptional activity through binding with TCF4.

Phosphomimetic β-catenin induces metastasis in an *in vivo* mouse model, upregulating CD44, TSG6, laminin γ2, and c-Jun. PLK1-mediated phosphorylation of β-catenin facilitates the interaction with TCF4/LEF. Binding TCF4, a component of the TCF4/LEF/β-catenin complex 31, with phosphomimetic β-catenin upregulates the expression of laminin γ2, CD44, TSG6, and c-Jun, which were highly upregulated in active-PLK1-driven EMT. Additionally, c-Jun, a component of AP-1, is also known as a transcriptional target of β-catenin in colorectal cancer 31 and is a possible transcriptional factor for PLK1 expression 32. The upregulated c-Jun activates the expression of PLK1 by direct binding of the PLK1 promoter, indicating that PLK1-mediated phosphorylation of β-catenin at Ser311 regulates *JUN* expression through TCF4 binding, and increased c-Jun upregulates the expression of PLK1 in a positive feedback loop. The expression of PLK1 by AP-1 is amplified by phosphorylated β-catenin through the direct expression of c-Jun. Therefore, PLK-mediated phosphorylation of β-catenin at Ser311 regulates its stability and its transcriptional activity connected by the axis of β-catenin/AP-1/PLK1 signaling.

Because laminin γ2, TSG6, and CD44 are components of ECM adhesion factors, their expression is directly connected with cancer cell migration and invasion for metastasis. Laminin γ2 is a subunit of glycoprotein laminin 322 that forms a web-like structure, providing tensile strength to the basement membrane in ECM 45. Laminin γ2 is identified as a metastatic marker with high expression in a number of cancers 45, including LUAD 23 and pancreatic ductal adenocarcinoma 46 because it enhances metastatic potential through integrin β1, ZEB1, and SNAI1-mediated EMT 23. In addition, TSG6 is involved in ECM stability during EMT and cell migration as an HA binding protein, a main component of proteoglycans in the ECM, which is a central factor for the EMT of renal tubular epithelial cells in a CD44-dependent manner 25, 26. CD44 is a cell adhesion molecule that works as a main transmembrane receptor for HA in ECM. As a regulatory mechanism for cell adhesion and migration, cells use HA to assemble microtentacles that can extend tens of micrometers from the cell body and adhere to the matrix via CD44 47. Based on studies, CD44 is established as a cancer stem cell marker and a mediator of metastasis 27, 47. Due to the functional importance of laminin γ2, TSG6, and CD44 in cancer migration and invasion as components of ECM, understanding their regulatory mechanism is important in the development of therapeutic strategies. In PLK1-driven EMT and metastasis, they function as main factors of metastasis in ECM 20. Especially, the expression or depletion of TSG6 is closely related to cancer invasion and the expression of EMT markers 20. Therefore, the phosphorylation of β-catenin by PLK1 is an important event in the upregulation of laminin γ2, CD44, and TSG6 in the ECM, which is a dynamic microenvironment in metastasis.

Clinically, PLK1 is established as a prognostic marker and a therapeutic target for the majority of solid, blood, and metastatic cancers, including gastric cancer 19, NSCLC 20, colorectal cancer 48, and prostate cancer 49. This is because PLK1 is recognized as a cause of malignancy and advanced cancer development 16, 17. The correlation between the levels of PLK1 and poor prognosis is well studied in most cancers including NSCLC 16, 17. Moreover, the expression of PLK1 can be useful in the diagnosis of patients with a high risk of metastasis, because the levels of PLK1 are higher in patients with metastatic cancer than in patients with primary cancer 17. Therefore, PLK1 is a useful index of malignancy and advanced metastatic cancer for diagnostics, prognostics, and therapeutics in clinics. In addition, the clinical significance of β-catenin expression has been studied in several cancers including colorectal cancer 50, 51, NSCLC 52, 53, hepatocellular cancer 54, 55, and breast cancer 56, 57. Because it functions as a transcriptional factor, the levels of PLK1 in the nucleus are an important index for the prognosis and diagnosis of cancer. In primary and metastatic colorectal adenocarcinomas, nuclear staining for β-catenin is positive, indicating that nuclear staining for β-catenin is recognized as an important parameter for cancer diagnoses 50, 51. Whereas in NSCLC patients, the levels of cytoplasmic and nuclear β-catenin are all correlated with poor prognosis 52. Large-scale studies have addressed the clinical significance of the levels and subcellular locations of β-catenin relative to tumor progression, survival, and differential diagnosis in cancer 50, 52. In this study, the clinical relevance between the expression of both PLK1 and β-catenin and survival rates of patients with NSCLC revealed that high *CTNNB1/PLK1* expression is inversely correlated with the survival rates, especially in metastatic NSCLC (Figure 1). Therefore, the high expression of *CTNNB1* and* PLK1* could serve as important diagnostic and prognostic markers for metastatic NSCLC. Especially, p-β-catenin at Ser311 is important in maintaining its stability and transcriptional activity in metastatic NSCLC, which is useful in the diagnosis of NSCLC.

Understanding and regulating the key factors of metastasis are needed for therapeutic improvement in cancer and to increase the survival rates of patients with NSCLC. Thus, regulating the activity and expression of PLK1 and/or β-catenin would provide diagnostic, prognostic, and therapeutic strategies in NSCLC, especially metastatic NSCLC.

## Supplementary Material

Supplementary figures and tables.

## Abbreviations

ECM: extracellular matrix
EMT: epithelial-mesenchymal transition
DTT: dithiothreitol
HA: hyaluronan
HR: hazard ratio
KM plot: *Kaplan Meier* plot
LEF: lymphocyte enhancer factor
LUAD: lung adenocarcinoma
NSCLC: non-small cell lung cancer
OS: overall survival
PLK1: polo-like kinase 1
TCGA: The Cancer Genome Atlas
TCF: T-cell factor
TNFAIP6: TNF Alpha Induced Protein 6
