# Supplementary material for: PLK1-mediated phosphorylation of β-catenin enhances its stability and transcriptional activity for extracellular matrix remodeling in metastatic NSCLC
Source: Theranostics. 2023 Feb 5;13(3):1198–216. doi: 10.7150/thno.79318 (PMC9925311; doi:10.7150/thno.79318)
Supplement: Supplementary file 1 — Supplementary figures and tables. [file thnov13p1198s1.pdf]

Supplementary Information

PLK1-mediated phosphorylation of β-catenin enhances its stability and transcriptional activity for extracellular matrix remodeling in metastatic NSCLC

Da-Eun Kim[1], Sol-Bi Shin[1], Chang-Hyeon Kim[1], Yeo-Bin Kim[1], Hyun-Ji Oh[1], and Hyungshin Yim[1,2 *]

[1]Department of Pharmacy, College of Pharmacy, Hanyang University, Ansan, Gyeonggi-do 15588, Korea

[2]Institute of Pharmaceutical Science and Technology, Hanyang University, Ansan, Gyeonggi-do 15588, Korea

[*]Corresponding author

Hyungshin YIM

Address: Department of Pharmacy, College of Pharmacy, Institute of Pharmaceutical Science and Technology, Hanyang University, Ansan, Gyeonggi-do 15588, Korea

Phone: +82-31-400-5810

Fax: +82-31-400-5958

E-mail: hsyim@hanyang.ac.kr

Supplementary Figure S1

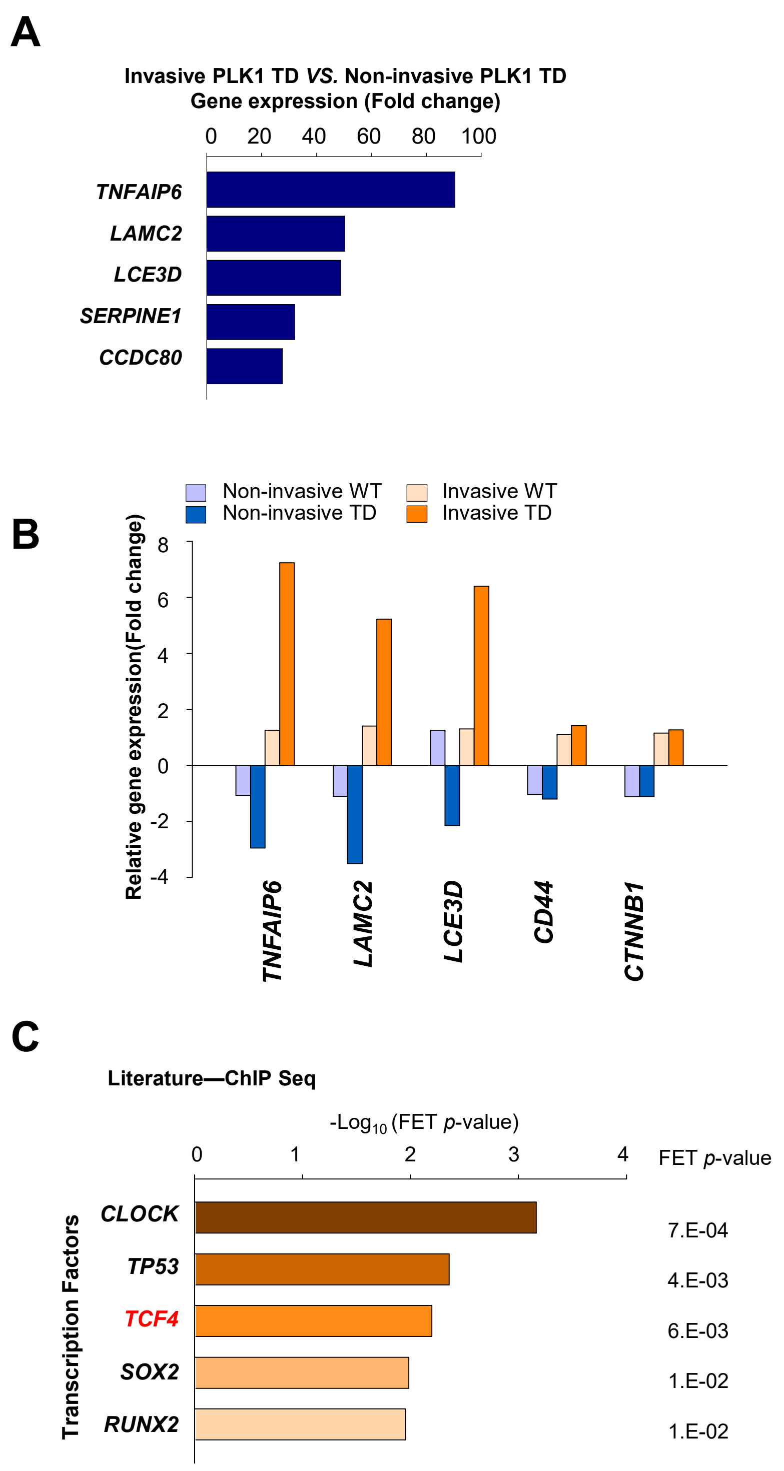

Supplementary Figure S1. Upregulation of ECM adhesion factors in active PLK1-driven EMT. A-B, A549 cells expressing wild-type (WT) or a constitutively active version of PLK1 (T210D; TD) were cultured in a three-dimensional Transwell [1]. **A,** Reanalysis of the relative gene expression profile of the top five genes was analyzed, normalized, and plotted in invasive A549 cells expressing TD mutant of PLK1 *vs.* non-invasive A549 cells expressing TD mutant of PLK1, using previously published microarray data [1]. **B,** Reanalysis of relative gene expression levels of the *TNFAIP6, LAMC2, LCE3D, CD44*, and *CTNNB1* in invasive and non-invasive cells expressing WT and TD, respectively, using previously published microarray data [1]. **C,** Prediction of transcriptional factors of top 30 genes in the invasive TD/non-invasive TD of PLK1 [1] was performed, and the top five were extracted from APPYTER.

Supplementary Figure S2

A

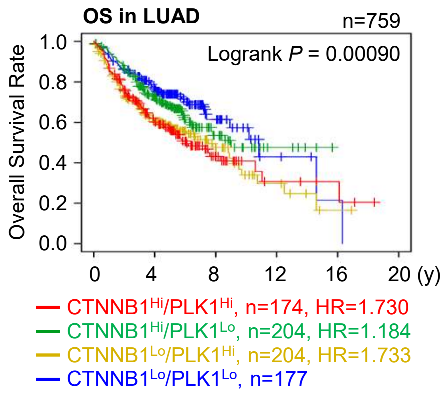

B

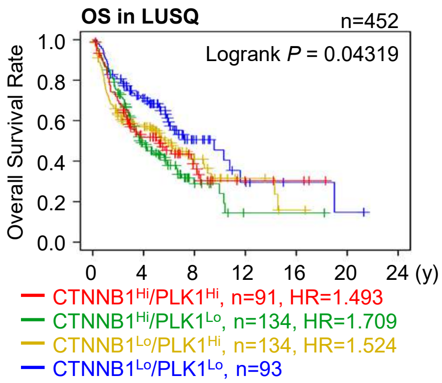

Supplementary Figure S2. Relevance of PLK1 and β-catenin in LUAD and LUSQ.

A-B, The overall survival (OS) times in LUAD patients (*n*=759, Log-rank *P* = 0.00090) (**A**) and LUSQ patients (n=452, Log-rank *P* = 0.04319) (**B**), were analyzed according to their *PLK1* and *CTNNB1* expression levels. High (Hi) and low (Lo) were generated by dividing patients according to their expression at the median cut-off.

Supplementary Figure S3

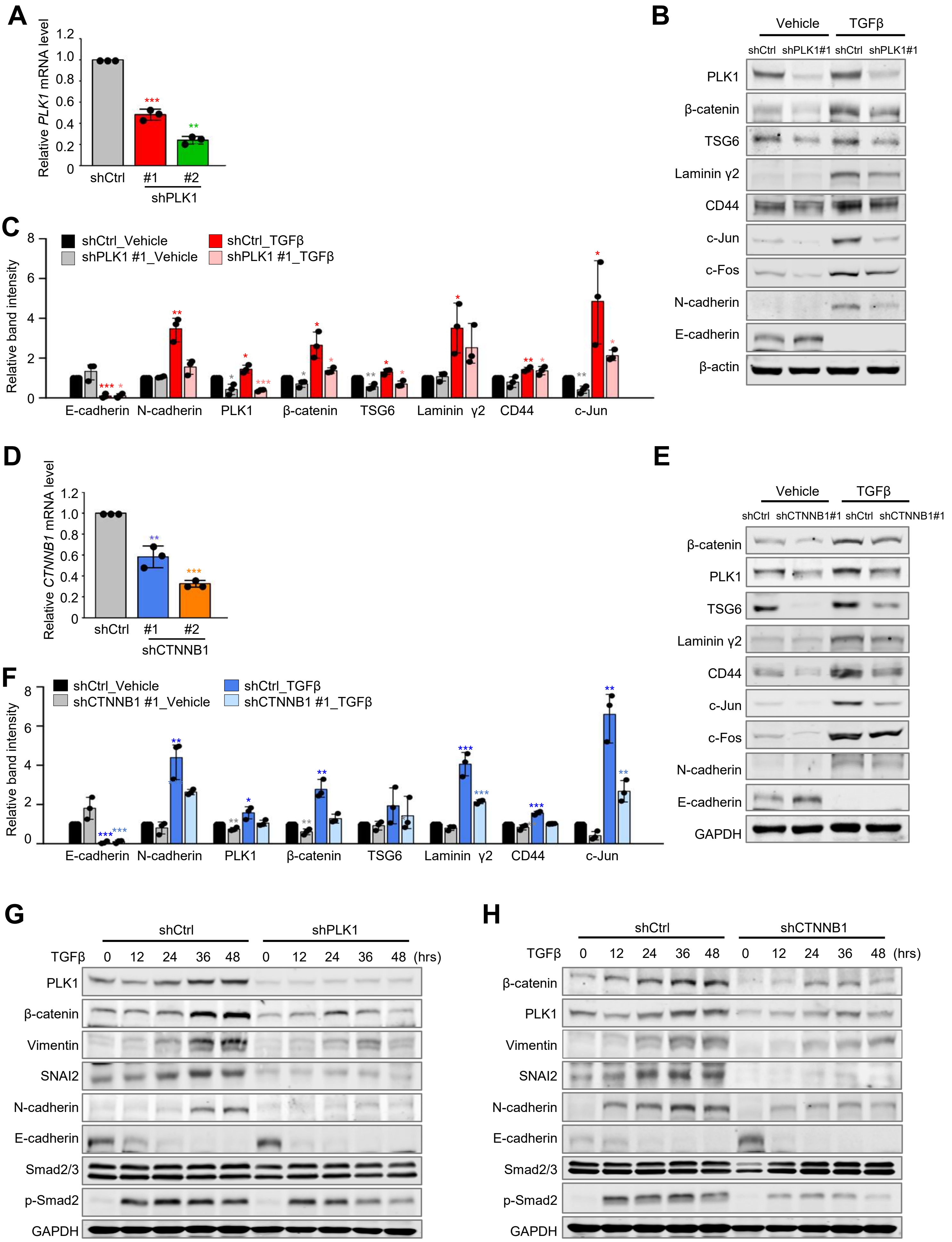

Supplementary Figure S3. Depletion of PLK1 or β-catenin downregulates the expression of TSG6, laminin γ2, CD44, and N-cadherin in TGF-β-induced EMT. A, PLK1-depleted cells using specific shRNAs targeting human *PLK1* (shPLK1 #1 and #2). qRT-PCR was performed for *PLK1* expression in A549 cells with depleted PLK1. **B-C,** Immunoblotting was performed using specific antibodies for PLK1, β-catenin, TSG6, laminin γ2, CD44, c-Jun, c-Fos, N-cadherin, E-cadherin, and β-actin. The band intensity values were using densitometry of Photoshop software, normalized, and plotted (**C**) *p <0.05; **p <0.01; ***p <0.001; (n=3). Data are presented as mean ± SD. **D,** β-catenin-depleted cells using specific shRNAs targeting human *CTNNB1* (shCTNNB1 #1 and #2). qRT-PCR was performed for *CTNNB1* expression in A549 cells with depleted *CTNNB1*. **E-F,** Immunoblotting was performed using specific antibodies for β-catenin, PLK1, TSG6, laminin γ2, CD44, c-Jun, c-Fos, N-cadherin, E-cadherin, and GAPDH. The band intensity values were using densitometry of Photoshop software, normalized, and plotted (**F**) *p <0.05; **p <0.01; ***p <0.001; (n=3). Data are presented as mean ± SD. **G-H,** PLK1- or β-catenin-depleted cells using specific shPLK1 # 2 or shCTNNB1 #2 were treated with TGF-β in A549 cells for 48 hours. Cells were prepared in a time-dependent manner at the indicated time. Immunoblotting was performed using specific antibodies for PLK1, β-catenin, vimentin, SNAI2, N-cadherin, E-cadherin, p-Smad2, Smad2/3, and GAPDH.

Supplementary Figure S4

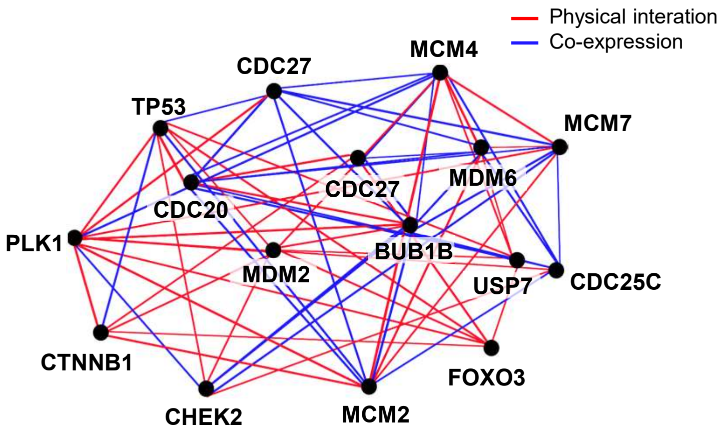

Supplementary Figure S4. The interactome analysis for PLK1 and β-catenin. The interactome analysis for PLK1 and β-catenin, which were extracted from GeneMANIA.

Supplementary Figure S5

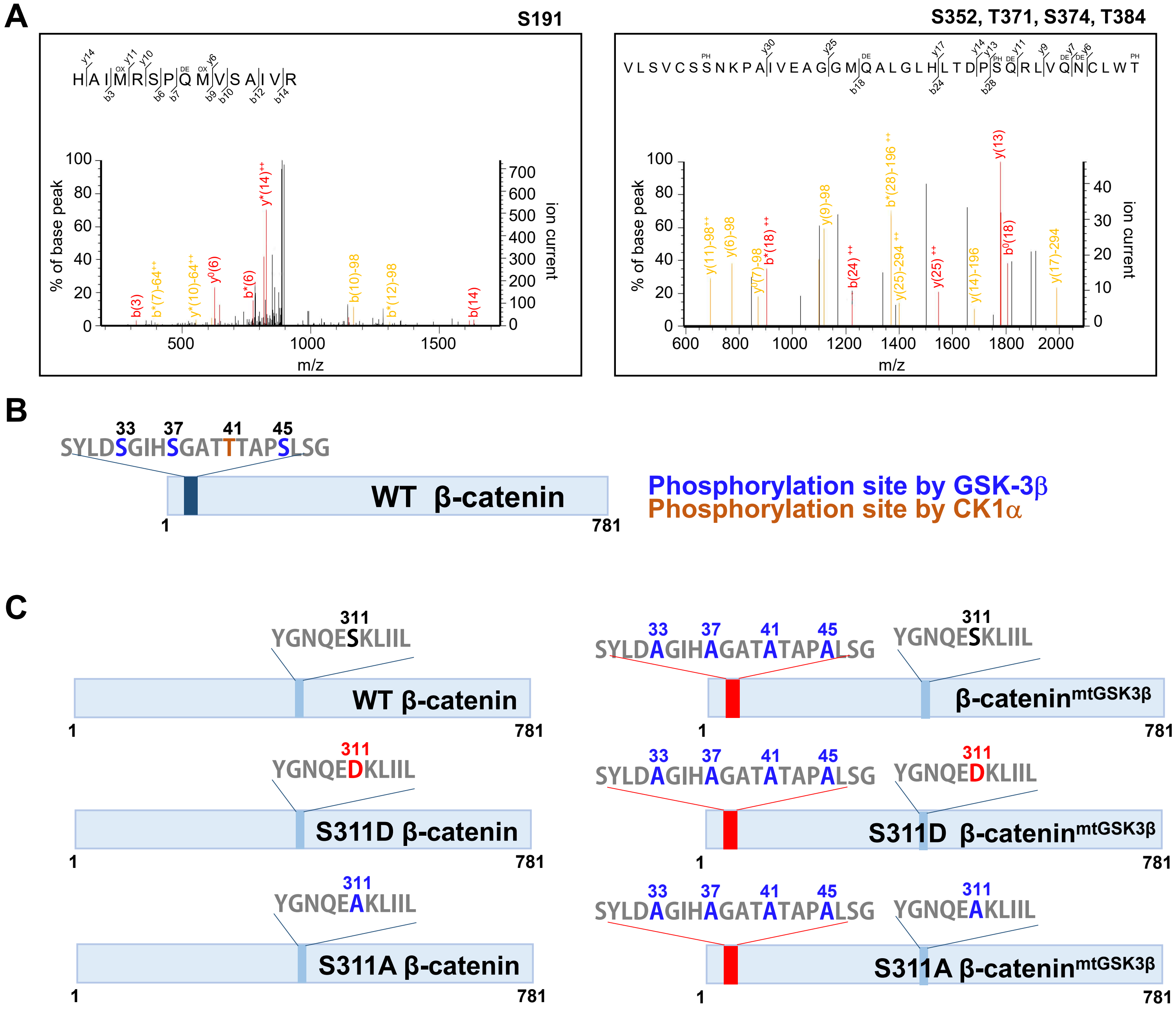

D

SPECIES	MOTIF
Homo sapiens	306 YGNQESKLIIL 316
Pan Troglodytes	306 YGNQESKLIIL 316
Macaca mulatta	306 YGNQESKLIIL 316
Canis lupus familiaris	296 YGNQESKLIIL 306
Mus musculus	306 YGNQESKLIIL 316
Xenopus tropicalis	292 YGNQESKLIIL 302
Rattus norvegicus	306 YGNQESKLIIL 316
Danio rerio	305 YGNQESKLIIL 315
Dorsophila melanogaster	314 YGNQESKLIIL 324

Supplementary Figure S5. Phosphorylation of β-catenin by PLK1. A, In the LC-MS/MS analysis, possible phosphorylation residues of β-catenin by PLK1 were detected at S191, T298, S311, S352, T371, S374, and T384. The LC/MS-MS data for T298 and S311 are shown in Figure 3E. **B,** The phosphorylation residues of β-catenin by GSK-3β and CK1α [2, 3]. **C,** The scheme of various version of β-catenin and β-catenin$^{mtGSK3\beta}$ structure. **D,** The plausible phosphorylation residues of β-catenin by PLK1 at Ser311 is evolutionarily conserved in several species.

Supplementary Figure S6

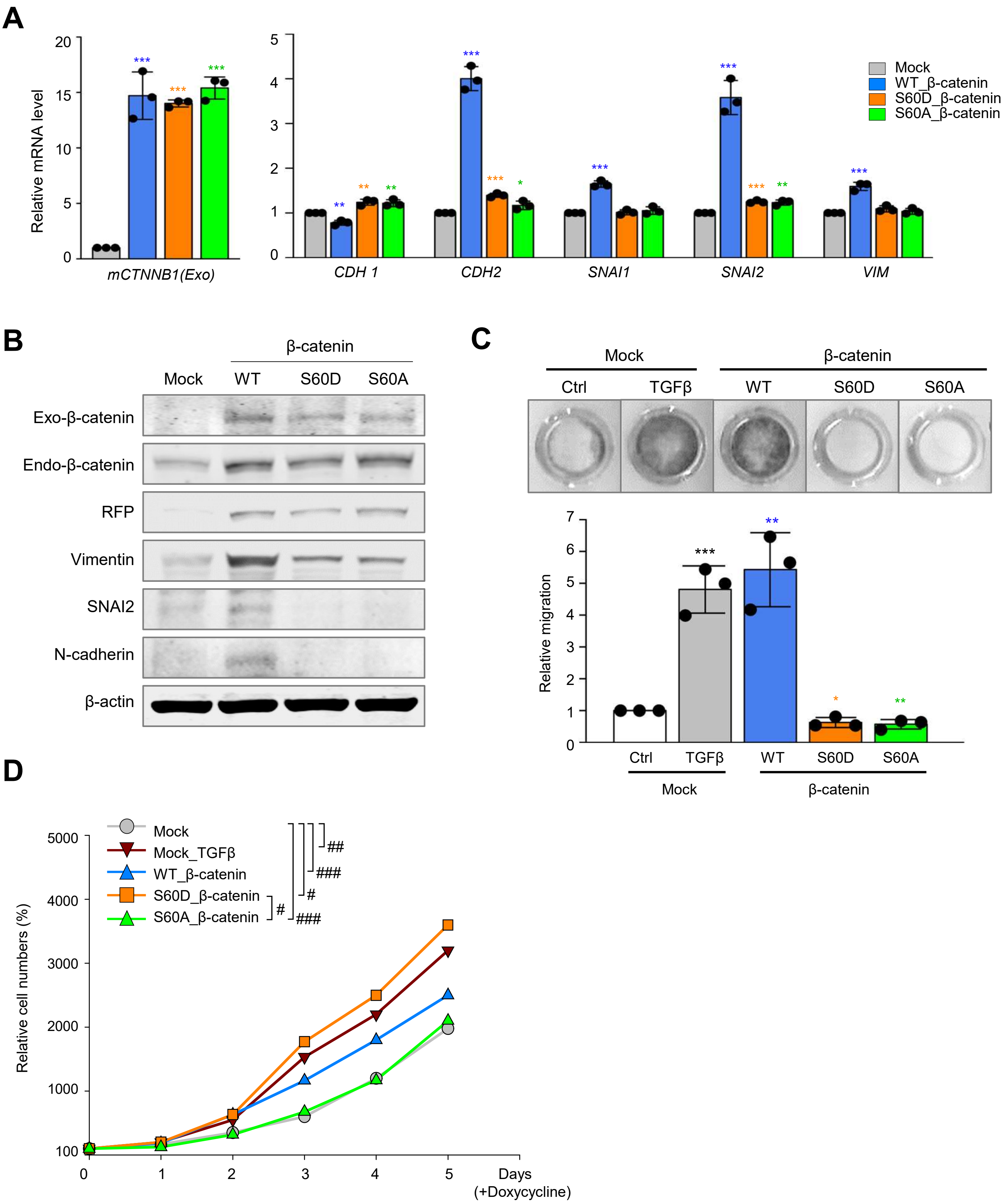

Supplementary Figure S6. Phosphorylation of β-catenin at Ser60 did not regulate EMT. The A549 cell expressing RFP-tagged WT, S60D, S60A of β-catenin was expressed for 48 hours using a doxycycline-inducible system. A549 cells were treated with doxycycline to express RFP-tagged β-catenin. **A,** qRT-PCR was performed for *CTNNB1*, *CDH1, CDH2*, *SNAI1, SNAI2*, and *VIM* in A549 cells expressing wild-type or mutant β-catenin. *p <0.05; **p <0.01; ***p <0.001; (n=3). Data are presented as mean ± SD. **B,** Immunoblotting was performed using specific antibodies for β-catenin, RFP, vimentin, SNAI2, N-cadherin, and β-actin. The band intensity values were quantified using the densitometry of Photoshop software, normalized, and plotted. **C,** Cells expressing wild-type or mutants of β-catenin were subjected to a Transwell migration assay. As a positive control for migration, cells were treated with TGF-β. Three days after seeding, the cells on the bottom layer surface were stained with 0.05% crystal violet dye. Images of the Transwell cell migration assay were collected and analyzed with an Odyssey infrared imaging system (LI-COR Biosciences) and plotted. *p <0.05; **p <0.01; ***p <0.001 compared with experimental control. **D,** Cell proliferation assay was performed (n=3). Data are presented as mean ± SD of at least three independent experiments. #, p < 0.05; ##, p <0.01; ###, p <0.001 compared with indicated groups of cells.

Supplementary Figure S7

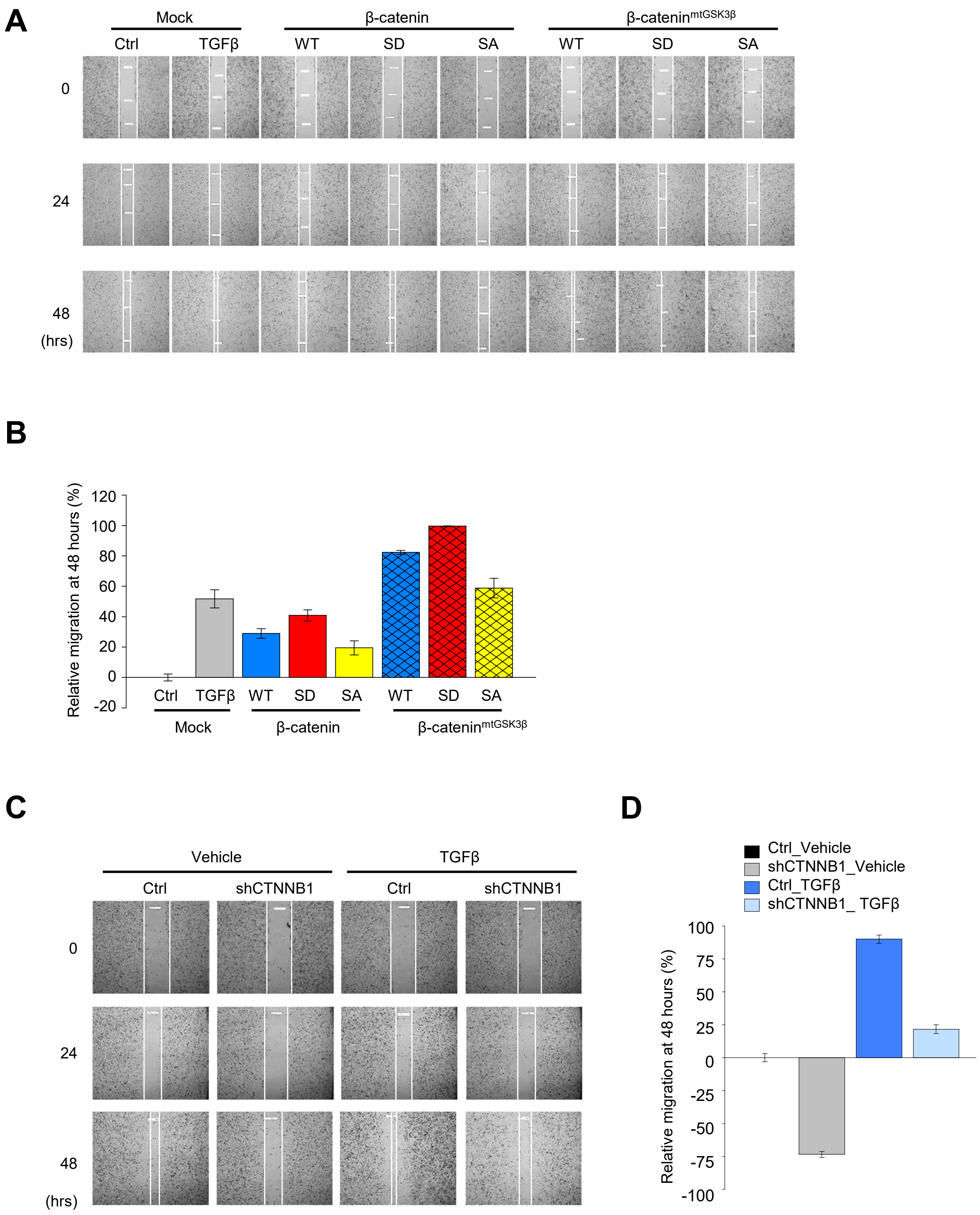

Supplementary Figure S7. Phosphorylated β-catenin promotes cell mobility of NSCLC. A-B, RFP-tagged wild-type (WT), S311D, or S311A of β-catenin and WT, S311, S311D, or S311A of β-catenin$^{mtGSK3\beta}$ (S33/S37/T41/S45A) mutants were expressed in A549 cells. A549 cells were treated with doxycycline to express RFP-tagged β-catenin and subjected to a wound healing assay. **A**, The scratch recovery efficiency after 48 hours was analyzed using NIS-Elements Imaging software (Nikon, Japan). **B**, The relative migration distance was plotted compared with the control. Data presented as mean ± SD. **C-D,** A549 cells were infected by lentiviral β-catenin shRNA (shCTNNB1 #2) and then treated with TGF-β for 48 hours. **C,** The scratch recovery efficiency after 48 hours was analyzed using NIS-Elements Imaging software (Nikon, Japan). **D,** The relative migration distance compared with the control was plotted. Data presented as mean ± SD.

Supplementary Figure S8

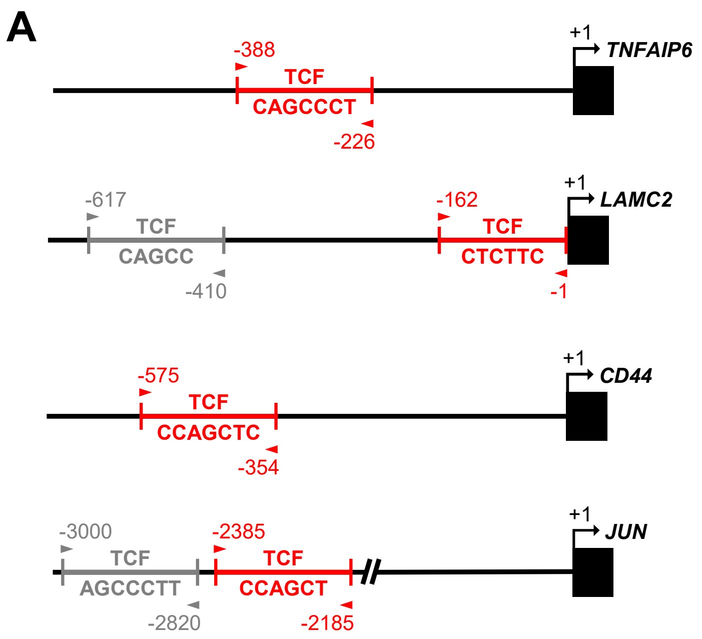

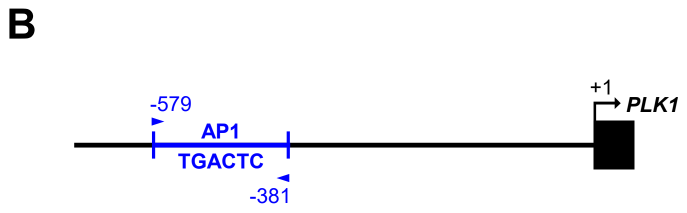

Supplementary Figure S8. Scheme of the promoter regions for ChIP assay.

A, Scheme of TCF4 binding regions in the promoter of *LAMC2, CD44, JUN,* and *TNFAIP6*. Based on TCF-binding elements characterized by a highly conserved consensus sequence with 5′-C(G/C)AGC(T/C)CTTC-3′ [4, 5], the TCF4 binding regions were displayed in the promoter of *LAMC2, CD44, JUN,* and *TNFAIP6*. **B,** Scheme of c-Jun binding regions in the promoter of *PLK1*. Based on AP-1-binding elements (5′-TGAG/CTCA-3’) [6], the AP-1 binding regions were displayed in the promoter of PLK1.

Supplementary Tables

Supplementary Table S1. Correlation between PLK1 and CTNNB1 expression and prognosis in various clinicopathological subsets of NSCLC.

Patients	Clinicopathological Factors	Gene expression	Number of patients (n)	Hazard Ratio (HR)	95% Confidential interval (CI)	*p*-Value
NSCLC	Whole data	$CTNNB1^{Hi}/PLK1^{Hi}$	272	1.590	1.243 – 2.035	0.00023
		$CTNNB1^{Hi}/PLK1^{Lo}$	372	1.276	1.005 – 1.620	0.04508
		$CTNNB1^{Lo}/PLK1^{Hi}$	371	1.601	1.268 – 2.022	7.68e-05
		$CTNNB1^{Lo}/PLK1^{Lo}$	277	-	-	-
Stage	1	$CTNNB1^{Hi}/PLK1^{Hi}$	105	2.143	1.316 – 3.49	0.00218
		$CTNNB1^{Hi}/PLK1^{Lo}$	155	1.621	1.01 – 2.603	0.04541
		$CTNNB1^{Lo}/PLK1^{Hi}$	155	1.872	1.175 – 2.982	0.00828
		$CTNNB1^{Lo}/PLK1^{Lo}$	107	-	-	-
	3 and 4	$CTNNB1^{Hi}/PLK1^{Hi}$	9	5.346	1.392 – 20.524	0.0146
		$CTNNB1^{Hi}/PLK1^{Lo}$	16	2.759	0.777 – 9.798	0.1165
		$CTNNB1^{Lo}/PLK1^{Hi}$	16	1.738	0.482 – 6.267	0.3984
		$CTNNB1^{Lo}/PLK1^{Lo}$	7	-	-	-
Histology	LUAD	$CTNNB1^{Hi}/PLK1^{Hi}$	174	1.730	1.232 – 2.430	0.00157
		$CTNNB1^{Hi}/PLK1^{Lo}$	204	1.184	0.835 – 1.679	0.34270
		$CTNNB1^{Lo}/PLK1^{Hi}$	204	1.733	1.243 – 2.417	0.00119
		$CTNNB1^{Lo}/PLK1^{Lo}$	177	-	-	-
	LUSQ	$CTNNB1^{Hi}/PLK1^{Hi}$	91	1.493	0.991 – 2.249	0.05537
		$CTNNB1^{Hi}/PLK1^{Lo}$	134	1.709	1.173 – 2.489	0.00528
		$CTNNB1^{Lo}/PLK1^{Hi}$	134	1.524	1.040 – 2.232	0.03069
		$CTNNB1^{Lo}/PLK1^{Lo}$	93	-	-	-

Supplementary Table S2. Sequences of forward (F) and reverse (R) primers used for RT-PCR amplification.

Target Gene	Primer	Sequences
Human *CTNNB1*	Forward<br>Reverse	5'- CCTTGGATATCGCCAGGA - 3'<br>5'- GCAGCCCATCAACTGGAT - 3'
Mouse *Ctnnb1*	Forward<br>Reverse	5'- CCTTGGATATCGCCAGGA - 3'<br>5'- GCAGCCCATCAACTGGAT - 3'
Human *CDH1*	Forward<br>Reverse	5'- ACCACCTCCACAGCCACC - 3'<br>5'- GTCCAGTTGGCACTCGCC - 3'
Human *CDH2*	Forward<br>Reverse	5'- ACAGTGGCCACCTACAAAGG - 3'<br>5'- CCGAGATGGGGTTGATAATG - 3'
Human *TNFAIP6*	Forward<br>Reverse	5'- GTGGCGTCTTTACAGATCC - 3'<br>5'- CATCTCCACAGTATCTTCCC - 3'
Human *LAMC2*	Forward<br>Reverse	5'- GCCTTTTGGCACCTGTATTC - 3'<br>5'- CAGGATTCTCATCCCCTGAA - 3'
Human *CD44*	Forward<br>Reverse	5'- CTGAGCATCGGATTTGAGAC - 3'<br>5'- CATACTGGGAGGTGTTGGAT - 3'
Human *PLK1*	Forward<br>Reverse	5'- AAGAGATCCCGGAGGTCCTA - 3'<br>5'- TCATTCAGGAAAAGGTTGCC - 3'
Mouse *Plk1*	Forward<br>Reverse	5'- CGAGGATCTGGAGGTGAAAA - 3'<br>5'- TCTCTTTTAGGCACGAGGTC - 3'
Human *JUN*	Forward<br>Reverse	5'- ATCCTGAAACAGAGCATGAC - 3'<br>5'- GTTGCTGGACTGGATTATCA - 3'
Human *VIM*	Forward<br>Reverse	5'- GAGAACTTTGCCGTTGAAGC - 3'<br>5'- GCTTCCTGTAGGTGGCAATC - 3'
Human *SNAI1*	Forward<br>Reverse	5'- GGAAGCCTAACTACAGCGAG - 3'<br>5'- CAGAGTCCCAGATGAGCATTG -3 '
Human *SNAI2*	Forward<br>Reverse	5'- ACGCCCAGCTACCCAATG - 3'<br>5'- AGGGCGCCCAGGCTCACATA - 3'
Human *ZEB1*	Forward<br>Reverse	5'- TGGGATCAACCACCAATGG - 3'<br>5'- AAGTAACCCTGTGTATTTCTGGATGA - 3'
Human *TWIST*	Forward<br>Reverse	5'- GGACAAGCTGAGCAAGATTCAGA - 3'<br>5'- TCTGGAGGACCTGGTAGAGGAA - 3'
Human *GAPDH*	Forward<br>Reverse	5'- TAAAGGGCATCCTGGGCTACACT - 3'<br>5'- TTACTCCTTGGAGGCCATGTAGG - 3'
Mouse *Gapdh*	Forward<br>Reverse	5'- GTTGTCTCCTGCGACTTCA - 3'<br>5'- GGTGGTCCAGGGTTTCTTA - 3'

Supplementary Table S3. Sequences of forward (F) and reverse (R) primers used for site-directed mutagenesis of mouse β-catenin.

Target Residue	Primer	Sequences
S191A	Forward<br>Reverse	5'- ATGCCATCATGCGCGCCCCTCAGATGGTG- 3'<br>5'- CACCATCTGAGGGGCGCGCATGATGGCAT- 3'
T298A	Forward<br>Reverse	5'- GTGAAATTCTTGGCTATTACAGCAGACTGCCTTCAGATC- 3'<br>5'- GATCTGAAGGCAGTCTGCTGTAATAGCCAAGAATTTCAC- 3'
S311A	Forward<br>Reverse	5'- AGCTTATGGCAATCAAGAGGCCAAGCTCATCATTCTGGCC - 3'<br>5'- GGCCAGAATGATGAGCTTGGCCTCTTGATTGCCATAAGCT - 3'
S311D	Forward<br>Reverse	5'- AGCTTATGGCAATCAAGAGGACAAGCTCATCATTCTGGCC - 3'<br>5'- GGCCAGAATGATGAGCTTGTCCTCTTGATTGCCATAAGCT - 3 '
S352A	Forward<br>Reverse	5'- GCTGTCTGTCTGCTCTGCCAACAAGCCGGCCATT- 3'<br>5'- AATGGCCGGCTTGTTGGCAGAGCAGACAGACAGC- 3'
T371A	Forward<br>Reverse	5'- CTGGGGCTTCATCTGGCAGACCCAAGTCAGC- 3'<br>5'- GCTGACTTGGGTCTGCCAGATGAAGCCCCAG- 3'
S374A	Forward<br>Reverse	5'- GGCTTCATCTGACAGACCCAGCTCAGCGACTTGTTCAA- 3'<br>5'- TTGAACAAGTCGCTGAGCTGGGTCTGTCAGATGAAGCC- 3'
T384A	Forward<br>Reverse	5'- CTTGTTCAAAACTGTCTTTGGGCTCTCAGAAACCTTTCAGATG- 3<br>5'- CATCTGAAAGGTTTCTGAGAGCCCAAAGACAGTTTTGAACAAG- 3''
S60A	Forward<br>Reverse	5'- GGAAGAAGATGTTGACACCGCCCAAGTCCTTTATGAATG - 3'<br>5'- CATTCATAAAGGACTTGGGCGGTGTCAACATCTTCTTCC - 3'
S60D	Forward<br>Reverse	5'- AGGAAGAAGATGTTGACACCGACCAAGTCCTTTATGAATGGG - 3'<br>5'- CCCATTCATAAAGGACTTGGTCGGTGTCAACATCTTCTTCCT - 3'

Supplemental Table S4. Sequences of forward (F) and reverse (R) primers used for ChIP assay.

Target Gene	Primer	Sequences
Human *LAMC2*	Forward<br>Reverse	5' - ATTTTCCAGCCCGGTTTG - 3'<br>5' - GCCTTCCTTTTCCTTGATCAG - 3'
Human *CD44*	Forward<br>Reverse	5' - AGAATGAGCTCTCCCTCTTTC - 3'<br>5' - ACCATTGGGTTCAGCCTTTG - 3'
Human *JUN*	Forward<br>Reverse	5' - AAGCGCTATTTCCTCTGCAG - 3'<br>5' - CCCACAACTGTCTTGAGAGAC - 3'
Human *TNFAIP6*	Forward<br>Reverse	5' - CTTGTTCAGTGCAGCCCTAT - 3'<br>5' - ACTAGCTGAAAACCCAGCAA - 3'
Human *PLK1*	Forward<br>Reverse	5' - AGGCCCTGGGAAATTCAG - 3'<br>5' - GCCATCACCTGAGAGCTT- 3'

References

1. Shin, S.B., Jang, H.R., Xu, R., Won, J.Y. & Yim, H. Active PLK1-driven metastasis is amplified by TGF-beta signaling that forms a positive feedback loop in non-small cell lung cancer. *Oncogene* **39**, 767-785 (2020).
2. Liu, C. *et al.* Control of beta-catenin phosphorylation/degradation by a dual-kinase mechanism. *Cell* **108**, 837-847 (2002).
3. van Noort, M., Meeldijk, J., van der Zee, R., Destree, O. & Clevers, H. Wnt signaling controls the phosphorylation status of beta-catenin. *J Biol Chem* **277**, 17901-17905 (2002).
4. Chang, Y.W. *et al.* Diverse Targets of beta-Catenin during the Epithelial-Mesenchymal Transition Define Cancer Stem Cells and Predict Disease Relapse. *Cancer Res* **75**, 3398-3410 (2015).
5. van de Wetering, M., Oosterwegel, M., Dooijes, D. & Clevers, H. Identification and cloning of TCF-1, a T lymphocyte-specific transcription factor containing a sequence-specific HMG box. *EMBO J* **10**, 123-132 (1991).
6. Shaulian, E. & Karin, M. AP-1 as a regulator of cell life and death. *Nat Cell Biol* **4**, E131-136 (2002).
